# Exploring Host-driven Immunopathological Factors Developing Severe Tuberculosis: Insights from Comparative Mouse Models

**DOI:** 10.7150/ijbs.124878

**Published:** 2026-02-04

**Authors:** Hongmin Kim, Kee Woong Kwon, Hagyu Kim, WeonSeok Jung, Kyungmin Kim, Jung Joo Hong, Sung Jae Shin

**Affiliations:** 1 Department of Microbiology, Institute for Immunology and Immunological Disease, Yonsei University College of Medicine, Seoul, South Korea.; 2 Department of Microbiology and Convergence of Medical Science, College of Medicine, Gyeongsang National University, Jinju, South Korea.; 3 Department of Microbiology, Graduate School of Medical Science, Brain Korea 21 Project, Yonsei University College of Medicine, Seoul, South Korea.; 4 Department of Microbiology, Graduate School of Medicine, Yonsei University College of Medicine, Seoul, South Korea.; 5 National Primate Research Center, Korea Research Institute of Bioscience and Biotechnology (KRIBB), Cheongju, Chungcheongbuk, South Korea.; 6 KRIBB School of Bioscience Korea University of Science & Technology (UST), Daejeon, South Korea.

**Keywords:** *mycobacterium tuberculosis*, host susceptibility, neutrophil-to-T cell ratio, type I interferon, inbred mouse models

## Abstract

Tuberculosis (TB) pathogenesis arises from complex interactions between host immune responses and the genetic diversity of *Mycobacterium tuberculosis* (Mtb). To elucidate host determinants of TB immunopathology, we conducted a comparative analysis of inbred mouse strains infected with the highly virulent Mtb K strain. Among the strains tested, C3H/HeJ and A/J mice exhibited markedly increased susceptibility, characterized by elevated pulmonary bacterial burdens and extensive necrotizing lung pathology. Interestingly, at 2 weeks post-infection (PI), both strains showed lower bacterial burdens, limited dissemination, and less pulmonary inflammation than C57BL/6 mice, but at 4 weeks PI, this trend reversed. The increased disease severity was closely associated with pronounced pulmonary neutrophilic infiltration, elevated systemic levels of granulocyte colony-stimulating factor (G-CSF), expansion of Lin⁻Sca-1⁻c-Kit⁺CD34⁺CD16/32⁺ granulocyte-monocyte progenitors (GMPs) in the bone marrow (BM), and a substantially increased pulmonary neutrophil-to-T cell (N/T) ratio, which positively correlated with disease progression. Depletion of neutrophils or blockade of type I IFN from 2 weeks PI significantly ameliorated disease severity, as evidenced by reduced bacterial burden, improved lung pathology, and normalization of the N/T ratio. Notably, IL-10 receptor blockade and aging specifically mitigated disease severity in A/J mice, whereas BCG vaccination conferred greater protection in C3H/HeJ mice. These strain-specific protective effects were consistently associated with restored N/T ratios, normalized GMP levels, and attenuated systemic G-CSF levels. Together, our findings identify the pulmonary N/T ratio and GMP expansion as central, mechanistically linked drivers of type I IFN signaling and neutrophil-mediated TB immunopathology.

## Introduction

Tuberculosis (TB) continues to be a global leading cause of morbidity and mortality among infectious diseases, accounting for 1.23 million deaths in 2024 (2025 WHO Report). Infection with Mtb, the causative agent of TB, presents as a spectrum of conditions that range from asymptomatic subclinical infection (LTBI) to active TB (ATB). However, the classic dichotomy between active and latent disease has been continuously revisited with dynamic spectrum extension 1-3. The long-standing view that Mtb infection exists in a simple binary state of either ATB or LTBI is increasingly recognized as an outdated oversimplification. Instead, Mtb infection is now understood to exist along a broad clinical and immunological continuum 4, 5. This spectrum encompasses individuals who remain uninfected despite repeated exposure (“resisters”); those who become infected but subsequently eliminate the pathogen; individuals with asymptomatic and stable infection; persons harboring latent infection with high risk of reactivation; patients with chronic, symptomatic ATB; and those who progress to rapidly progressive and severe disease. Over the past decade, this nuanced concept has gained wider acceptance, largely driven by advances in transcriptional profiling, *in vivo* imaging, and more sophisticated clinical studies, which together have provided deeper insights into TB pathogenesis 3. The TB spectrum is shaped by the dynamic interplay between host immune responses and the burden imposed by Mtb 2, 6.

Severe TB immunopathology accompanied by cavitation with a substantial burden of Mtb and extensive tissue destruction represents the most hazardous form of TB and serves as a direct cause of mortality. This uncontrolled TB often leads to unfavorable consequences such as lengthy treatment duration with increased drug toxicity, poor treatment outcomes, higher relapse post-treatment, higher transmission rates, and the development of drug resistance 7-10. Moreover, the majority of relapses and treatment failures are associated with cavitary TB 7, 8. In addition, even after successful treatment with microbiological cure, sequelae of lung damage and destruction may develop serious long-term lung impairments, called post-TB lung disease 11-15.

Findings from multiple post-mortem investigations indicate that human pulmonary cavities tend to arise in areas of lipoid pneumonia, which are regions of lung inflammation caused by the accumulation of lipid-rich material, rather than in well-structured granulomas, a point that has often been underappreciated 16. However, fundamental mechanistic research in this area is severely lacking. In the antibiotic era, cavities have often been regarded as the most severe manifestation of treatment failure and remain among the least explored features of TB 8. The development of cavities through the liquefaction of caseum is considered a key process driving both the transmission and persistence of the disease. Individuals with cavities may harbor bacterial burdens reaching up to 10^11^ bacilli per gram of tissue, which makes them highly infectious. Collectively, as a result of the action of pathological host responses, including profound productions of proinflammatory cytokines, chemokines, accumulated immune cells, and alterations in lipid species that mediate TB-associated necrosis and extracellular matrix destruction in the lung may result in a higher tendency of TB immunopathology 17, 18.

Accumulating evidence from animal experiments and clinical studies revealed the type I interferon (IFN) signaling and neutrophilic inflammation were shown to correlate with disease severity of TB 19-24. For example, Berry et al. demonstrated that ATB correlated with a neutrophil-driven type I IFN signature in blood, suggesting extent of lung disease and the disease progression from latent TB sate 24. For another example, Eum et al. demonstrated that neutrophils were the most abundant cell type containing Mtb in ATB patients, even in cavity caseum sites 19. In animal studies, Kotov et al. recently proposed that type I IFN production by plasmacytoid dendritic cells in response to neutrophil extracellular trap (NET) caused uncontrolled bacterial replications in Mtb-susceptible *Sp140^-/-^* mice 20. In addition, genetically TB-susceptible C3HeB/FeJ mice representing human-like TB granulomas exhibited type I IFN-induced NET formation and uncontrolled neutrophil-mediated lung inflammation that promotes bacterial growth and disease severity 22. Moreover, Kang et al. also demonstrated that viral coinfection-induced type I IFN signaling generated TB immunopathology with an increased neutrophil population in Mtb-resistant C57BL/6J mice 21. Taken together, excessive neutrophil infiltration at the site of infection and the activation of type I IFN response are consistently associated with TB exacerbation clinically and experimentally. While the mechanism underlying type I IFN signaling and neutrophil-mediated tissue pathology have been extensively studied in respect with innate immune regulations, a quantitative assessment of the complex roles in adaptive immunity and cellular composition has been poorly understood in this detrimental process of TB immunopathology.

We previously reported that C3H/HeJ mice, which carry a loss-of-function mutation in TLR4 25 and A/J mice, which lack complement component C5 26, both exhibit severe pulmonary inflammation and poor bacterial control following Mtb infection. In present study, we expanded how neutrophils and type I IFN response in both TB-susceptible mice acts as common immunopathological drivers of TB by comparing with TB-resistant C57BL/6J mice which has strong Th1-biased response 27, in particular on the side of neutrophil-to-T cell (N/T) ratios, granulocyte-monocyte progenitors (GMPs), aging, and BCG vaccine effect. Our findings indicate that both strains serve as valuable models for studying the TB spectrum heterogeneity, as they share common pathological mechanisms under diverse immunological conditions.

## Materials and Methods

### Animals and ethics statement

All animal procedures were performed in accordance with the institutional guidelines and national regulations established by the Korea Centers for Disease Control and Prevention (KCDC) and the Korean Food and Drug Administration (KFDA). Experimental protocols were approved by the Institutional Animal Care and Use Committee (IACUC) and the Ethics Committee of the Laboratory Animal Research Center, Yonsei University College of Medicine, Seoul, Korea (Permit No. 2022-0012, 2020-0103, and 2016-0170). Female specific pathogen-free (SPF) mice (C57BL/6J, DBA/2, A/J, and C3H/HeJ), aged 6 weeks, were purchased from Japan SLC, Inc. (Shizuoka, Japan). Mice were housed under Animal Biosafety Level 3 (ABSL-3) conditions at the Avison Biomedical Research Center, Yonsei University College of Medicine. The facility maintained a controlled environment at 24 ± 1 °C with 50 ± 5% relative humidity and a 12-hour light/dark cycle (lights on at 07:00 h and off at 19:00 h). Animals had ad libitum access to sterile commercial feed and autoclaved water.

### Preparation of mycobacteria strains

Mtb K strain was obtained from the Korean Institute of Tuberculosis (KIT, Osong, Chungcheongbuk-do, Korea) and the Mtb H37Rv strain (ATCC 27294) was purchased from the American Type Culture Collection (ATCC, Manassas, VA, USA). *M. bovis* BCG (Pasteur 1173P2) was kindly provided by Dr. Brosch (Pasteur Institute, Paris, France). All mycobacterial strains were cultured in Middlebrook 7H9 broth supplemented with 10% oleic acid-albumin-dextrose-catalase (OADC; Difco Laboratories, Detroit, MI, USA), following standard procedures as described previously 28.

### Antibodies and reagents

Mouse recombinant granulocyte-macrophage colony-stimulating factor (GM-CSF) was obtained from Creagene (Seoul, Korea). Pam3CSK4 (Pam3) and CpG ODNs 1826 (ODNs) were purchased from InvivoGen (San Diego, California, USA).

### Mtb challenge and BCG immunization

Six-week-old mice were subjected to aerosol exposure with Mtb strains in a calibrated inhalation chamber (Glas-Col, Terre Haute, IN, USA) for 60 minutes, ensuring administration of a defined infectious dose. Unless otherwise indicated, mice were challenged with approximately 300 CFUs of Mtb K per animal. For the assessment of bacterial burden, organs were excised, homogenized, and the resulting homogenates were serially diluted and cultured on Middlebrook 7H10 agar plates (BD Biosciences, San Jose, CA, USA) containing amphotericin B and 10% OADC (Difco Laboratories, Detroit, MI, USA). Following incubation of the plates at 37 °C for 3 weeks, colonies were subsequently enumerated. Data are expressed as mean log_10_ CFU per organ. In specific experiments, 18- and 20-week-old mice were challenged with Mtb K. BCG immunization was performed by subcutaneous administration of BCG Pasteur 1173P2 at 1 × 10^6^ CFUs per mouse, followed by aerosol challenge with Mtb K ten weeks after vaccination.

### *In vivo* experiment and antibody interventions

To achieve *in vivo* depletion or blockade of the indicated targets, Mtb K-infected mice received intraperitoneal injections of 250 μg anti-Ly6G antibody (clone 1A8; Bio X Cell, Lebanon, NH, USA) and anti-IL-10 receptor antibody (clone 1B1.3 A; Bio X Cell, Lebanon, NH, USA) on days 14, 16, 18, 21, 23, and 25 post-infection (PI), and anti-IFNAR1 antibody (clone MAR1-5A3; Bio X Cell, Lebanon, NH, USA) on days 13, 15, and 17 PI. Rat IgG2a (2A3; Bio X cell), rat IgG1(HRPN; Bio X cell), and mouse IgG1(MOPC-21; Bio X cell) isotype control antibodies were administered to control mice in correspondence with the original antibodies.

### Mtb enumeration and lung histopathological assessment

Homogenized suspensions of lungs and spleens were plated onto Middlebrook 7H10 agar (BD Biosciences, San Jose, CA, USA) supplemented with 10% OADC enrichment medium, and bacterial loads were quantified as described previously 29. Lung tissues were prepared for histopathological analysis by fixation in 10% neutral-buffered formalin overnight, followed by paraffin embedding, sectioning (4-5 μm), and H&E staining. Quantification of lung inflammation was performed using ImageJ software (National Institutes of Health, USA) according to previously published procedures 30. Grayscale conversion of images was performed, in which inflamed areas appeared black and non-inflamed areas white, and quantification of inflamed tissue was carried out using ImageJ.

### Preparation of single cells

Single-cell suspensions were generated by mincing lungs into small pieces and incubating them in RPMI medium containing 0.1% collagenase type II (Worthington Biochemical, Lakewood, NJ, USA) for 30 minutes at 37°C in a shaking incubator. After enzymatic digestion, the samples were filtered using a 40-μm nylon mesh strainer, subjected to RBC lysis buffer treatment (Gibco, Thermo Fisher Scientific, Waltham, MA, USA) for 3 minutes, and washed with RPMI1640 medium supplemented with 2% FBS. Spleens were dissociated by pressing through a 40-μm cell strainer (BD Falcon, Franklin Lakes, NJ, USA), and RBCs were removed with ACK lysis buffer (Gibco, Thermo Fisher Scientific, Waltham, MA, USA). Bone marrow (BM) cells were isolated from the femurs and tibias of mice and subjected to RBC lysis for 3 minutes using the same buffer.

### Flow cytometry analysis

To analyze lung immune cell populations, single-cell suspensions were seeded at 5 × 10^5^ cells per well in 96-well round-bottom plates using PBS supplemented with 2% FBS, and stained with antibodies for 20 minutes at 4°C. The antibodies were diluted at a ratio of 1:300 for the staining; fluorochrome-conjugated antibodies against CD11c (HL3), Ly6G (1A8), CXCR4 (2B11) were obtained from BD Bioscience (San Jose, CA, USA); fluorochrome-conjugated antibodies against CD11b (M1/70), MHC class II (M5/114.15.2), CD90.2 (Thy1.2; 53-2.1), Siglec-F (E50-2440), CD64 (X54-5/7.1), PD-L1 (10F.9G2) were obtained from BioLegend (San Diego, CA, USA); fluorochrome-conjugated antibodies against F4/80 (BM8), and CD62L (MEL-14) were obtained from Invitrogen (Carlsbad, CA, USA); fluorochrome-conjugated antibody against CXCR2 (242216) was obtained from R&D system (Minneapolis, MN, USA). The dead cells were stained with the Live/Dead Fixable Stain Kit (Thermo Fisher, Waltham, MA, USA). To analyze neutrophil-to-T (N/T) ratio, the frequency of lung neutrophil from each group was divided to the frequency of lung T cell.

For BM progenitor analysis, single-cell suspensions were seeded at 5 × 10^5^ cells per well and stained with antibodies for 20 minutes at 4°C. The BM cells were strained at a 1:50 dilution; biotin lineage antibody cocktail (120-001-547, Miltenyi Biotec, Bergisch Gladbach, Germany), and fluorochrome conjugated anti-biotin antibody (Bio3-18E7) were obtained from Miltenyi Biotec. Next, the cells were strained at a 1:300 dilution; fluorochrome-conjugated antibodies against Sca-1 (D7), C-Kit (2B8), CD16/32 (93), CD34 (RAM34) were obtained from Invitrogen.

To assess intracellular cytokine production in lung T cells, 5 × 10^5^ cells were stimulated with ESAT-6 (1 μg/mL) for 12 hours in the presence of GolgiPlug and GolgiStop (BD Biosciences, San Jose, CA, USA). For surface staining, cells were incubated for 20 minutes at 4 °C with fluorochrome-conjugated antibodies at a 1:300 dilution. Antibodies specific for CD90.2 (Thy1.2) and CD4 were obtained from BioLegend. To exclude non-viable cells, samples were stained with the Live/Dead Fixable Stain Kit (BD Biosciences, San Jose, CA, USA). Intracellular staining was performed following surface staining using the BD Cytofix/Cytoperm fixation and permeabilization kit (BD Biosciences, San Jose, CA, USA) according to the manufacturer's protocol. After permeabilization, cells were incubated for 30 minutes at 4 °C with fluorochrome-conjugated antibodies at a 1:200 dilution. Antibodies against IFN-γ (clone XMG1.2) and TNF-α (clone MP6-XT22) were purchased from BD Biosciences (San Jose, CA, USA).

To assess the functional state of neutrophils, cells were stained for 20 minutes at 4 °C with fluorochrome-conjugated antibodies at a 1:300 dilution, including anti-CD11b (clone M1/70; BioLegend, San Diego, CA, USA), anti-Ly6G (clone 1A8; BD Biosciences, San Jose, CA, USA), and anti-CD101 (clone Moushi101; Invitrogen, Carlsbad, CA, USA). To exclude non-viable cells, samples were stained using the Live/Dead Fixable Stain Kit (BD Biosciences, San Jose, CA, USA). NET formation was evaluated by staining cells with an antibody specific for citrullinated histone H3 (CitH3; citrulline R2 + R8 + R17; clone RM1001, Abcam, Cambridge, UK), followed by incubation with Alexa Fluor™ 594-conjugated donkey anti-rabbit IgG (H+L) secondary antibody (Invitrogen, Carlsbad, CA, USA) for 30 minutes at 4 °C. Intracellular nitric oxide synthase 2 (NOS2) expression was determined after permeabilization by incubation with a fluorochrome-conjugated anti-NOS2 antibody (clone W16030C; BioLegend) for 30 minutes at 4 °C.

### Measurement of intracellular reactive oxygen species

The levels of neutrophil intracellular reactive oxygen species (ROS) were measured using the oxidation-sensitive fluorescent dye H2DCFDA (2′,7′-dichlorodihydrofluorescein diacetate; Invitrogen, Carlsbad, CA, USA). Four weeks PI, lungs were removed and prepared as a single-cell suspension. The cells were incubated with 10 μM H2DCFDA fluorescent probe at 37 °C for 30 minutes in the dark, washed twice with cold PBS, and immediately analyzed for ROS levels by flow cytometry.

### Immunofluorescence imaging

Paraffin-embedded lung samples were sectioned at a thickness of 4 μm. The sections were deparaffinized in xylene, followed by heat-induced antigen retrieval in citrate buffer (pH 6.0) at sub-boiling temperature for 20 minutes. After gradual cooling and washing with PBS, nonspecific binding was blocked by incubation with CAS-Block™ (Invitrogen, Carlsbad, CA, USA) for 1 hour at room temperature. The sections were subsequently incubated overnight at 4 °C with the following primary antibodies: anti-Ly6G (clone 1A8, Bio X Cell, Lebanon, NH, USA), and anti-CitH3 (citrulline R2 + R8 + R17; clone RM1001 Abcam, Cambridge, UK). Following washes, sections were incubated for 1 hour at room temperature with Alexa Fluor™ 594-conjugated donkey anti-rat IgG (H+L) and Alexa Fluor™ 647-conjugated goat anti-rabbit IgG (H+L) secondary antibody (Invitrogen, Carlsbad, CA, USA) for 1 hour at room temperature. Nuclei were counterstained with DAPI (1 μg/mL), followed by mounting with Vectashield mounting medium (Vector Laboratories, Newark, CA, USA). Fluorescence images were obtained using a Leica THUNDER Imager DMi8 fluorescence microscope with a 40× objective lens. Quantitative analysis of fluorescence intensity was performed using ImageJ software.

### Magnetic cell sorting for neutrophils and assessment of bacterial growth and T cell suppression

To isolate Ly6G^+^ cells, lung cell suspensions from Mtb-infected mice were incubated with anti-Ly6G antibodies conjugated with microbeads and purified through sequential LS MACS column separation (Miltenyi Biotec, Bergisch Gladbach, Germany). To assess bacterial loads in Ly6G^+^ cells, isolated populations from Mtb-infected mice were lysed with 0.05% Triton X-100 (Biosesang, Gyeonggi-go, Republic of Korea), and plated onto Middlebrook 7H10 agar for enumeration of bacterial colonies.

For suppression assays, spleen cells obtained from naive C57BL/6, A/J, and C3H/HeJ mice were incubated with anti-CD4 microbeads and purified by sequential passage through an LS MACS column (Miltenyi Biotec, Bergisch Gladbach, Germany). Following labeling with 2.5 μM CellTrace Violet (Life Technologies, Carlsbad, CA, USA) for 20 minutes at 37 °C, T cells were washed in RPMI supplemented with 10% FBS and cultured in 96-well plates coated with 1 μg/mL anti-CD3 and anti-CD28 (BD Biosciences, San Jose, CA, USA). Labeled CD4^+^ T cells (1 × 10^5^) were cocultured with Ly6G^+^ cells at different ratios in RPMI medium supplemented with 10% FBS for 72 hours.

### Cytokine quantification

Lung and spleen single-cell suspensions were incubated with ESAT-6 for 12 hours at 37 °C. Cytokine concentrations, including IFN-γ, IL-5, IL-17A (Invitrogen, Carlsbad, CA, USA), and IL-10 (BioLegend, San Diego, CA, USA), in the supernatants, and granulocyte colony-stimulating factor (G-CSF; R&D Systems, Minneapolis, MN, USA) in serum, were quantified using ELISA kits according to the manufacturer's protocols.

### *In vitro* bone marrow cell culture with GM-CSF

BM cells were isolated from the femurs and tibias of C57BL6/J, A/J, and C3H/HeJ mice and were re-suspended at 5 × 10^5^ cells/mL. The cells were cultured with the RPMI1640 media containing GM-CSF (20 ng/mL) with or without the Pam3 (50 ng/mL) or ODNs (50 ng/mL) to mimic inflammation environment. After 3 days, fresh conditioned media were added to the cultured cells. Six days from cell culture, the cells were harvested and analyzed with flow cytometry.

### Quantitative real-time PCR

Total RNA from lung tissues was extracted using TRIzol reagent (Thermo Fisher, Waltham, MA, USA). RNA concentrations were measured with a NanoDrop™ 2000 spectrophotometer (Thermo Fisher Scientific). cDNA was synthesized from 1 μg of RNA using the RNA-to-cDNA EcoDry Premix (Oligo dT) (Takara Bio Inc., Kusatsu, Shiga, Japan), and subjected to qPCR with TB Green Ex Taq II (Tli RNase Plus, ROX Plus) (Takara Bio Inc., Kusatsu, Shiga, Japan). Amplification was carried out on an Applied Biosystems StepOne Plus Real-Time PCR System (Thermo Fisher, Waltham, MA, USA) under the following cycling conditions: 95 °C for 30 seconds, followed by 40 cycles of 95 °C for 5 seconds and 62 °C for 30 seconds. Gene expression was normalized to *Actb*, and relative expression levels were calculated using the 2-ΔΔCt method. The primers used for qPCR were as follows: *Ifnb* forward, 5′-GATGACGGAGAAGATGCAGAAG-3′; and reverse, 5′-ACCCAGTGCTGGAGAAATTG-3′.

### Statistical analysis

Unpaired Student's *t*-tests were employed to evaluate differences between two groups, whereas one-way ANOVA followed by Tukey's post hoc test was applied for comparisons among three or more groups. Survival analyses were conducted using the log-rank (Mantel-Cox) test. All data were analyzed with GraphPad Prism version 8.00 (GraphPad Software, San Diego, CA, USA), with statistical significance defined as *p < 0.05, **p < 0.01, and ***p < 0.001.

## Results

### C3H/HeJ and A/J mice exhibited markedly TB disease severity among mouse strains tested

As previously reported, the genotype of Mtb is a critical determinant of host immune modulation and has a substantial impact on the clinical course of TB. Distinct Mtb strains exhibit variable virulence, immune evasion mechanisms, and pathological characteristics, contributing to differences in disease severity, transmissibility, and treatment response 31, 32. In our previous study 26, A/J mice were more susceptible than C57BL/6 mice to infection with the reference H37Rv strain (Lineage 4, Euro-American), as evidenced by higher bacterial loads and more severe lung inflammation. Given that the Mtb K strain (Lineage 2, a prevalent Beijing lineage in Korea) induces more severe TB, characterized by increased bacterial burden, enhanced lung pathology, and higher relapse rates in mice 33, we compared disease severity in A/J mice (Fig. S1A), which are susceptible to H37Rv infection 26. Consistent with our previous findings, H37Rv elicited characteristic TB immunopathology; however, infection with Mtb K resulted in more extensive lung pathology, a higher N/T ratio, and greater bacterial burden relative to the initial infectious dose (Fig. S1B-D). Based on these findings, Mtb K was selected for subsequent experiments (hereafter simply referred to as “Mtb”).

**Figure 1:**
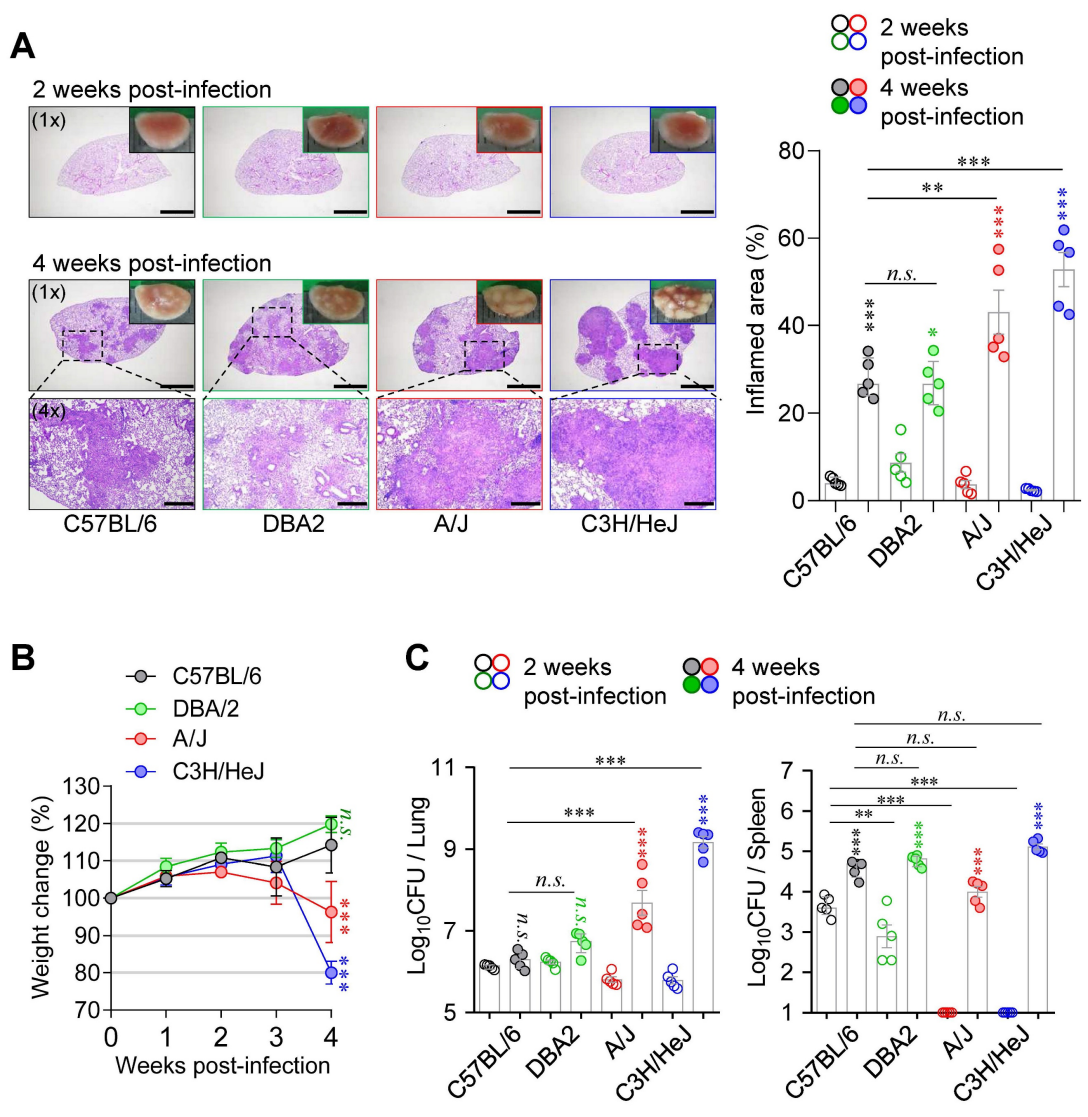
** Differential host susceptibility among inbred mouse strains following Mtb infection.** (A) Lung histopathology of four inbred mouse strains (C57BL/6, DBA/2, A/J, and C3H/HeJ) was analyzed using H&E staining at 2 and 4 weeks post-infection with Mtb K strain. Scale bar = 2 mm (1x), 0.5 mm (4x). *n* = 5. (B) The percentage of body weight changes were measured over 4 weeks post-infection in each mouse strain. *n* = 5. (C) Bacterial burdens in the lungs and spleens at 2 and 4 weeks post-infection were assessed. *n* = 5. Statistical analysis was performed by one-way ANOVA with Tukey's multiple comparisons. *n.s.* = not significant. **p < 0.01, ***p < 0.001. Undesignated asterisks indicate comparisons to the C57BL/6 at the same time point.

Next, to compare pathological responses during Mtb infection, six- to seven-week-old female C57BL/6J, DBA/2, A/J, and C3H/HeJ mice were infected with the Mtb via the aerosol route. Mice were sacrificed at 2- and 4-weeks PI to evaluate the severity of disease. At 2 weeks PI, A/J and C3H/HeJ mice exhibited lung Mtb burdens comparable to those of C57BL/6J and DBA/2 mice, but with markedly reduced lung inflammation (Fig. 1). Notably, both strains showed minimal bacterial dissemination to the spleen at this early time point (Fig. 1). However, this pattern was reversed by 4 weeks PI. A/J and C3H/HeJ mice developed pronounced TB immunopathology, characterized by necrotic lung lesions (Fig. 1A), significant body weight loss (Fig. 1B), and elevated pulmonary Mtb burdens (Fig. 1C). These findings indicate that C3H/HeJ and A/J mice exhibit the highest susceptibility to Mtb infection among the tested strains, ranking first and second, respectively, in terms of bacterial burden and lung pathology. Based on these findings, we selected C57BL/6 mice as a resistant strain and A/J and C3H/HeJ mice as moderately and highly susceptible strains, respectively, for subsequent experiments to characterize immunological features associated with TB susceptibility.

### Pulmonary neutrophil-to-T cell ratio positively correlated with TB-severity in different mouse strains during Mtb infection

**Figure 2:**
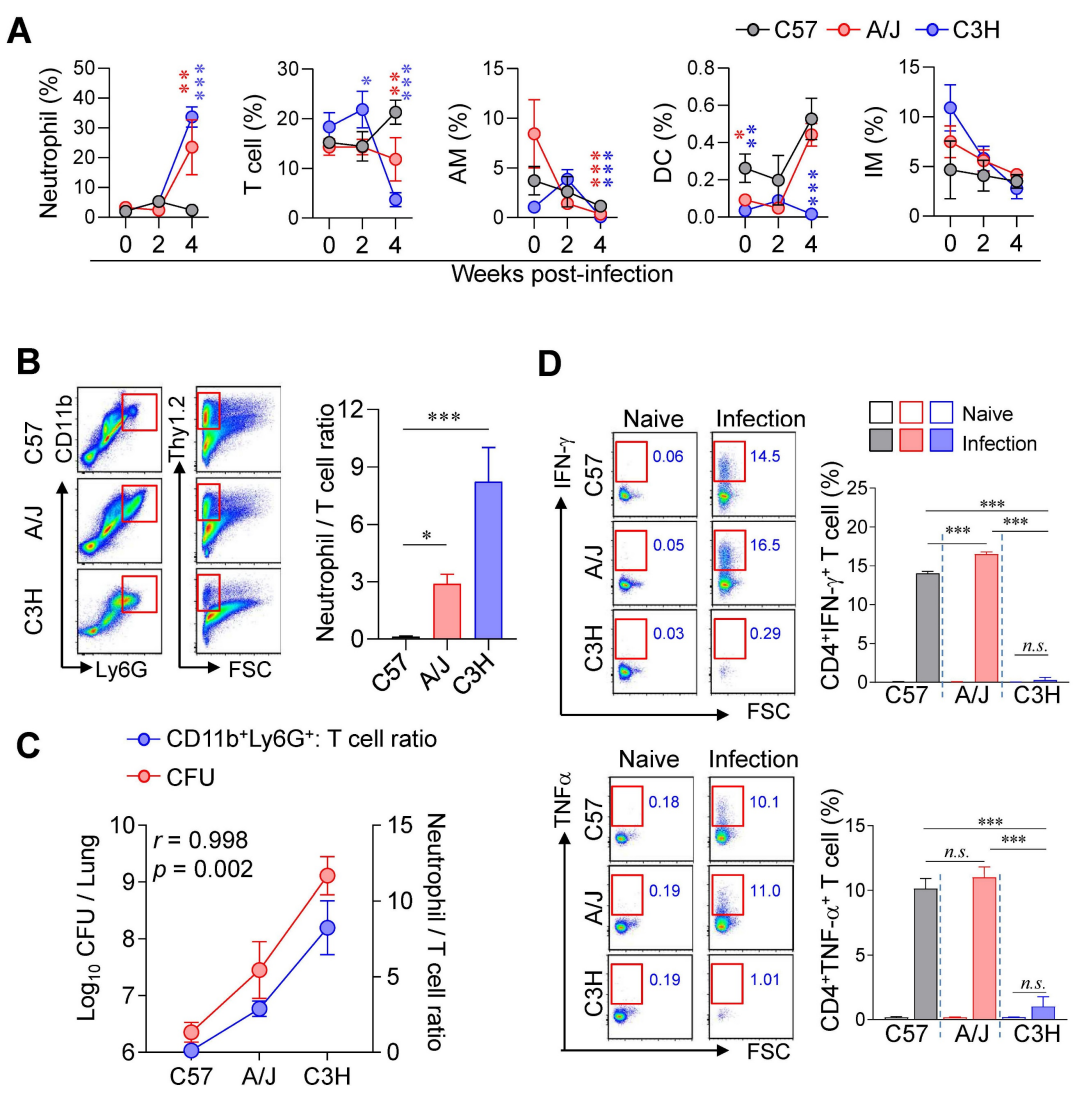
** Lung immune cell profiles associated with differential susceptibility to Mtb infection.** (A) The immune cell population of the lungs of C57BL/6, A/J, and C3H/HeJ mice was analyzed at indicated time point. *n* = 4 (B) Neutrophil-to-T cell ratio in the lungs at 4 weeks post-infection. *n* = 4. (C) Correlation between lung bacterial burden and N/T ratio at 4 weeks post-infection. *n* = 4. (D) Lung cells were stimulated with ESAT-6 to analyze IFN-γ- or TNF-α-producing CD4^+^ T cells. *n* = 4. Data are presented as mean ± SD. Statistical analyses were performed using one-way ANOVA with Tukey's multiple comparison test or Pearson correlation where applicable. *n.s.* = not significant, *p < 0.05, **p < 0.01, ***p < 0.001. AM, alveolar macrophage; DC, dendritic cell; IM, interstitial macrophage.

Next, we compared the lung immune cell composition of two TB-susceptible mouse strains, A/J and C3H/HeJ, with that of the TB-resistant C57BL/6J mice during Mtb infection using flow cytometric analysis (Fig. S2). Notably, both susceptible strains exhibited increased neutrophil infiltration and decreased T cell frequencies in the lungs compared to C57BL/6J mice (Fig. 2A, B). Furthermore, the N/T ratio strongly correlated with both lung CFU counts and the degree of TB susceptibility (Fig. 2C), suggesting that the N/T ratio is a potential indicator of susceptibility to TB. Interestingly, A/J mice showed antigen-specific Th1 responses and IFN-γ production comparable to C57BL/6J mice, whereas C3H/HeJ mice exhibited minimal Th1 responses and IFN-γ production throughout 2 to 4 weeks PI (Fig. 2D, Fig. S3). These findings indicate that A/J mice are characterized by an elevated N/T ratio despite preserved Th1 responses, while C3H/HeJ mice display the highest N/T ratio with an absence of antigen-specific Th1 responses. In sum, these results suggest that increased neutrophil infiltration, reduced T cell populations, and impaired antigen-specific Th1 responses are key determinants of TB susceptibility in mice.

### Neutrophil depletion significantly alleviated TB-immunopathology in both susceptible mice

**Figure 3:**
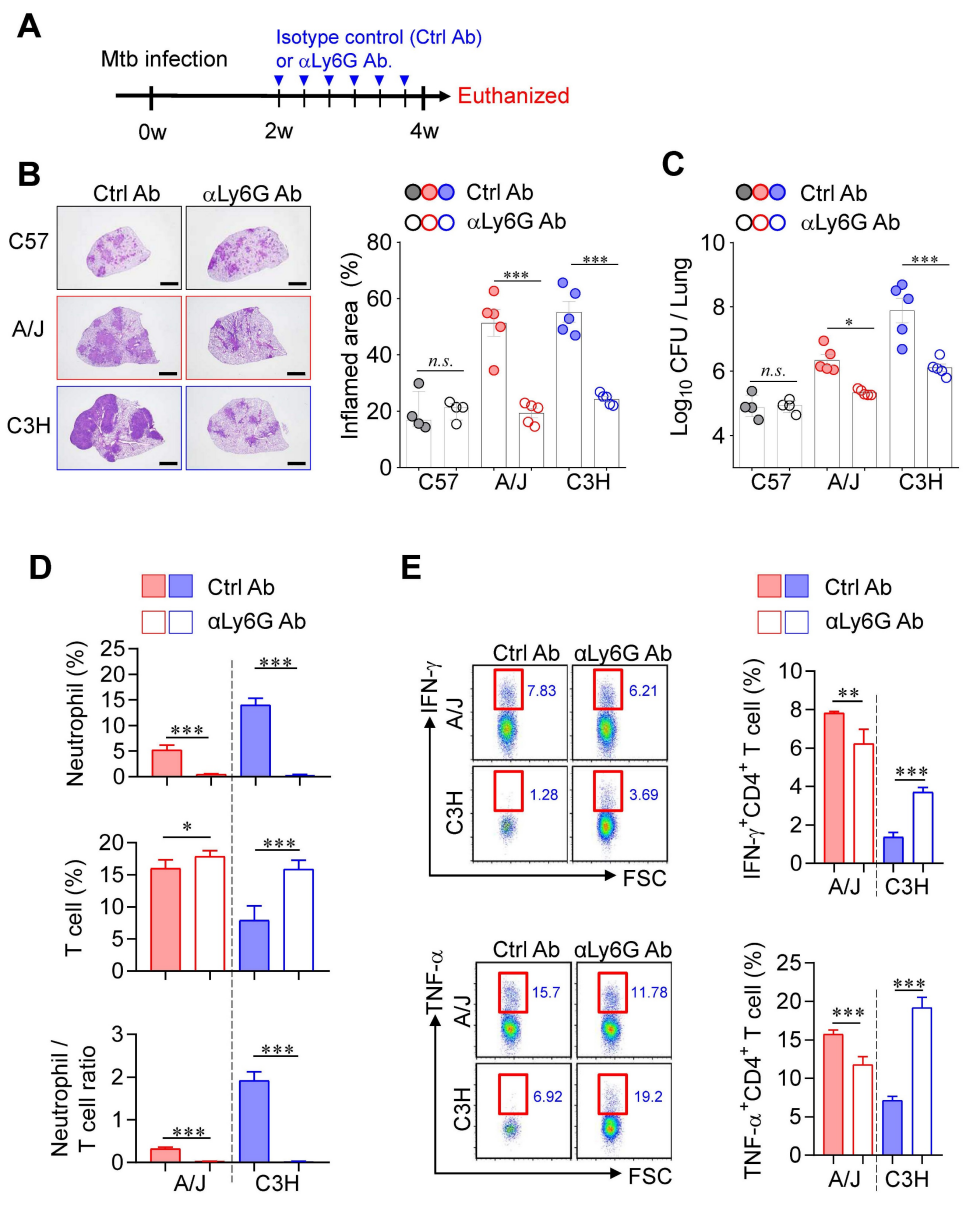
** Neutrophil depletion-mediated attenuated TB immunopathology in both TB-susceptible mouse strains.** (A) Each strain of mice was treated with anti-Ly6G antibody 3 times per week from 2 to 4 weeks post-infection to deplete neutrophils, followed by analysis at 4 weeks post-infection. (B) Lung histopathology was analyzed using H&E staining (scale bar = 2 mm), and (C) bacterial burdens in the lungs at 4 weeks post-infection were assessed. *n* = 4-5. (D) Neutrophil and T cell in the lungs were analyzed by flow cytometry at 4 weeks post-infection. *n* = 4-5. (E) Lung cells were stimulated with ESAT-6 to analyze IFN-γ or TNF-α producing CD4^+^ T cells. *n* = 4-5. Data are presented as mean ± SD. Statistical analyses were performed using one-way ANOVA with Tukey's multiple comparison test or Pearson correlation where applicable. *P < 0.05, **P < 0.01, ***P < 0.001.

Accumulating clinical and experimental evidence has demonstrated the involvement of neutrophils in TB infection, disease progression, severity, and mortality 34-37, suggesting that neutrophils may represent a promising target for host-directed therapy 38, 39. Moreover, IFN-γ produced by CD4⁺ T cells has been shown to directly suppress pathogenic neutrophil infiltration into the infected lungs 40, 41. To investigate the impact of neutrophils on TB immunopathology, we depleted neutrophils using an anti-Ly6G antibody (1A8) from 2 to 4 weeks PI in both TB-susceptible mouse strains (A/J and C3H/HeJ) (Fig. 3A). Strikingly, neutrophil depletion significantly suppressed TB progression in both susceptible strains, as evidenced by reduced lung inflammation and decreased Mtb burden (Fig. 3B, C), whereas no notable effects were observed in TB-resistant C57BL/6J mice. In parallel, T cell frequencies increased in both susceptible strains, with a particularly pronounced effect in C3H/HeJ mice, resulting in a significant reduction in the N/T ratio (Fig. 3D). Additionally, antigen-specific T cell responses were markedly enhanced, especially in C3H/HeJ mice by neutrophil depletion, while A/J mice showed only marginal changes (Fig. 3E). These findings suggest that neutrophils play a central role in driving TB immunopathology in TB-susceptible mouse strains and highlight their potential as a target for therapeutic intervention.

**Figure 4:**
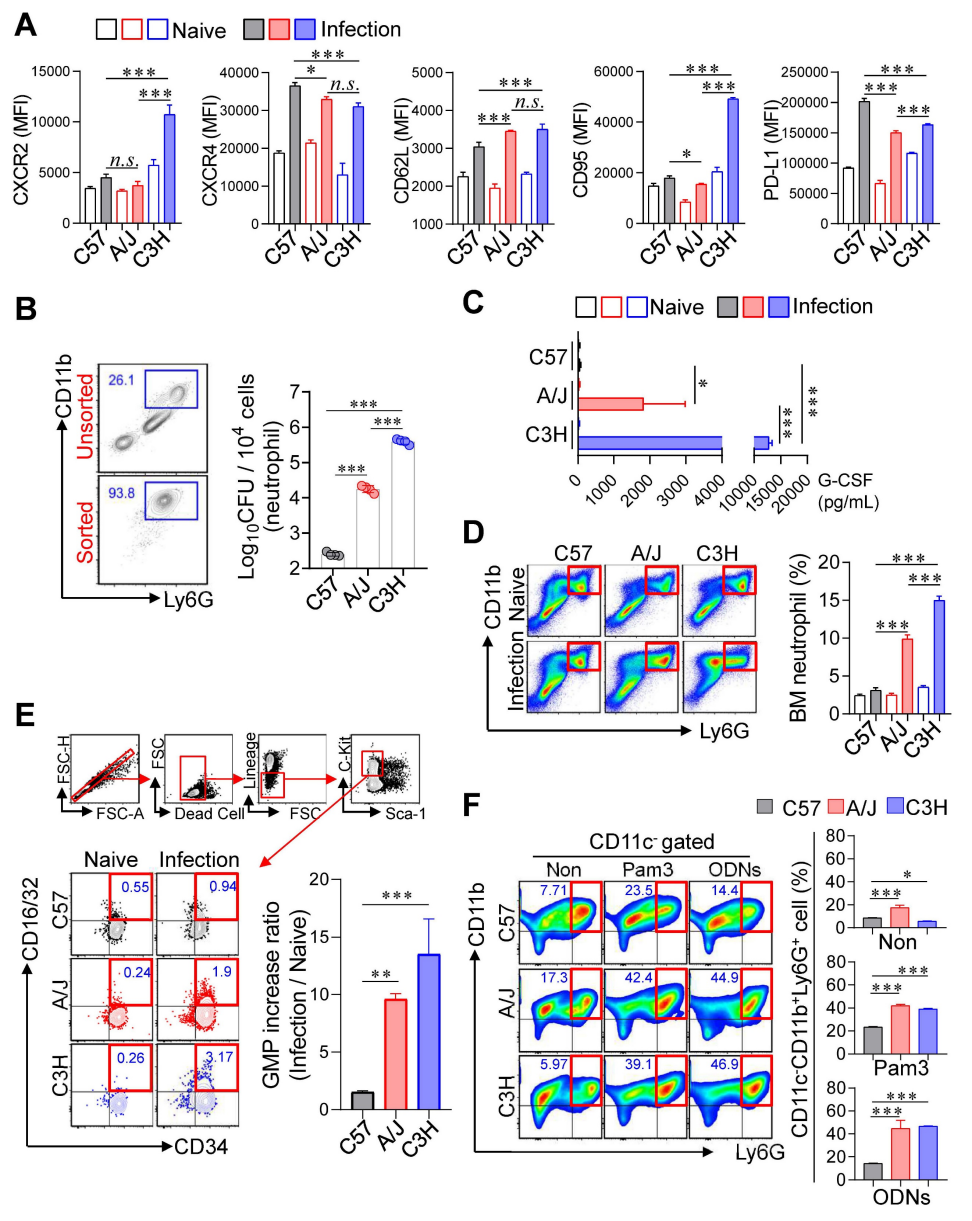
** Increased expression of type I IFN in the lungs of TB-susceptible inbred mouse strains.** (A) Surface molecules of the lung neutrophils from inbred mouse strains were analyzed by flow cytometry at 4 weeks post-infection. *n* = 4. (B) Bacterial burden of the isolated lung neutrophils was assessed. *n* = 4. (C) The level of G-CSF in serum was measured by ELISA. *n* = 3. At 4 weeks post-infection, the bone marrow cells were isolated from C57BL/6, A/J, and C3H/HeJ mice and (D) neutrophil and (E) GMP population was analyzed by flow cytometry. *n* = 3. (F) The bone marrow cells were cultured for 6 days with Pam3 or ODNs in the presence of GM-CSF, then the CD11c^-^CD11b^+^Ly6G^+^ population was analyzed with flow cytometry. *n* = 3. Data are presented as mean ± SD. Statistical analyses were performed using one-way ANOVA with Tukey's multiple comparison test. *P < 0.05, **P < 0.01, ***P < 0.001. GMP, granulocyte-monocyte progenitor.

Given the strong correlation between pulmonary neutrophil infiltration and TB severity, we next characterized the phenotype and function of neutrophils. The surface markers CD62L, CXCR2, CXCR4, CD95, and PD-L1 are associated with neutrophil maturation, inflammatory or immunosuppressive states, and regulation of adaptive immunity 42-47. We therefore analyzed the expression of these markers in neutrophils from TB-susceptible (C3H/HeJ and A/J) and TB-resistant (C57BL/6J) mice. CD62L, a marker of neutrophil maturation, was upregulated in TB-susceptible strains, whereas CXCR4 (linked to immaturity and inflammation) and PD-L1 (involved in T cell suppression) were downregulated. Especially, CXCR2 and CD95 (linked to immaturity and neutrophil infiltration) were upregulated in the neutrophil of C3H/HeJ mice, the most TB susceptible strain. This result suggested that a hyperinflammatory and functionally immature neutrophil phenotype in the TB-susceptible mice (Fig. 4A). We further assessed neutrophil permissiveness to Mtb by isolating pulmonary neutrophils from infected mice and quantifying intracellular bacterial loads. Neutrophils from C3H/HeJ and A/J mice exhibited significantly higher Mtb burden than those from C57BL/6J mice (Fig. 4B). As an initial functional assessment, we measured NOS2 expression and ROS production in pulmonary neutrophils. NOS2 expression was reduced, whereas ROS levels, NET formation, and CD101⁻ neutrophils, which have been previously implicated in type I IFN-mediated TB immunopathogenesis 48-51 were increased in neutrophils from TB-susceptible A/J and C3H/HeJ mice compared with C57BL/6 mice (Fig. S4A-E).

Building upon our previous finding that immature pathogenic lung neutrophils are induced by G-CSF in a NOX2^-/-^ mouse model following Mtb infection 36, we quantified serum G-CSF concentrations to evaluate their relationship with host susceptibility (Fig. 4C). This analysis revealed a significant positive correlation between serum G-CSF levels and TB susceptibility. To explore whether increased pulmonary neutrophils in susceptible strains reflected changes in BM output, we analyzed neutrophil and GMPs populations (Lineage^-^Sca-1^-^c-kit^+^CD34^+^CD16/32^+^) at 4 weeks PI. Both proportions of neutrophils and GMPs were significantly elevated in the BM of TB-susceptible mice compared to C57BL/6J (Fig. 4D, E), indicating enhanced granulopoiesis. Given that peripheral inflammation promotes neutrophil mobilization 52, we next examined whether BM cells from TB-susceptible mice preferentially differentiate into neutrophils under inflammatory conditions. BM cells were stimulated with GM-CSF, in the presence of Toll-like receptor (TLR) ligands, Pam3 for TLR2 or ODNs for TLR9, which mimic Mtb pathogen-associated molecular patterns 53, 54. Strikingly, BM cells from C3H/HeJ and A/J mice differentiated predominantly into CD11c⁻CD11b⁺Ly6G⁺ neutrophils upon stimulation, whereas C57BL/6J mice showed only marginal changes (Fig. 4F, Fig. S5A). Finally, we evaluated whether neutrophils from TB-susceptible mice function as myeloid-derived suppressor cells and inhibit Mtb-specific T cell responses 55. T cell suppression assays using pulmonary CD11b⁺Ly6G⁺ cells at 4 weeks PI revealed no significant differences in T cell suppression capacity among the strains when co-cultured with activated T cells (Fig. S5B). Taken together, these findings indicate that neutrophils in TB-susceptible mouse strains exhibit an immature, hyperinflammatory phenotype with increased Mtb permissiveness and enhanced granulopoietic potential, suggesting their central role in TB pathogenesis.

### Blockade of type I IFN signaling remarkably attenuated TB-immunopathology in both TB-susceptible mouse strains

**Figure 5:**
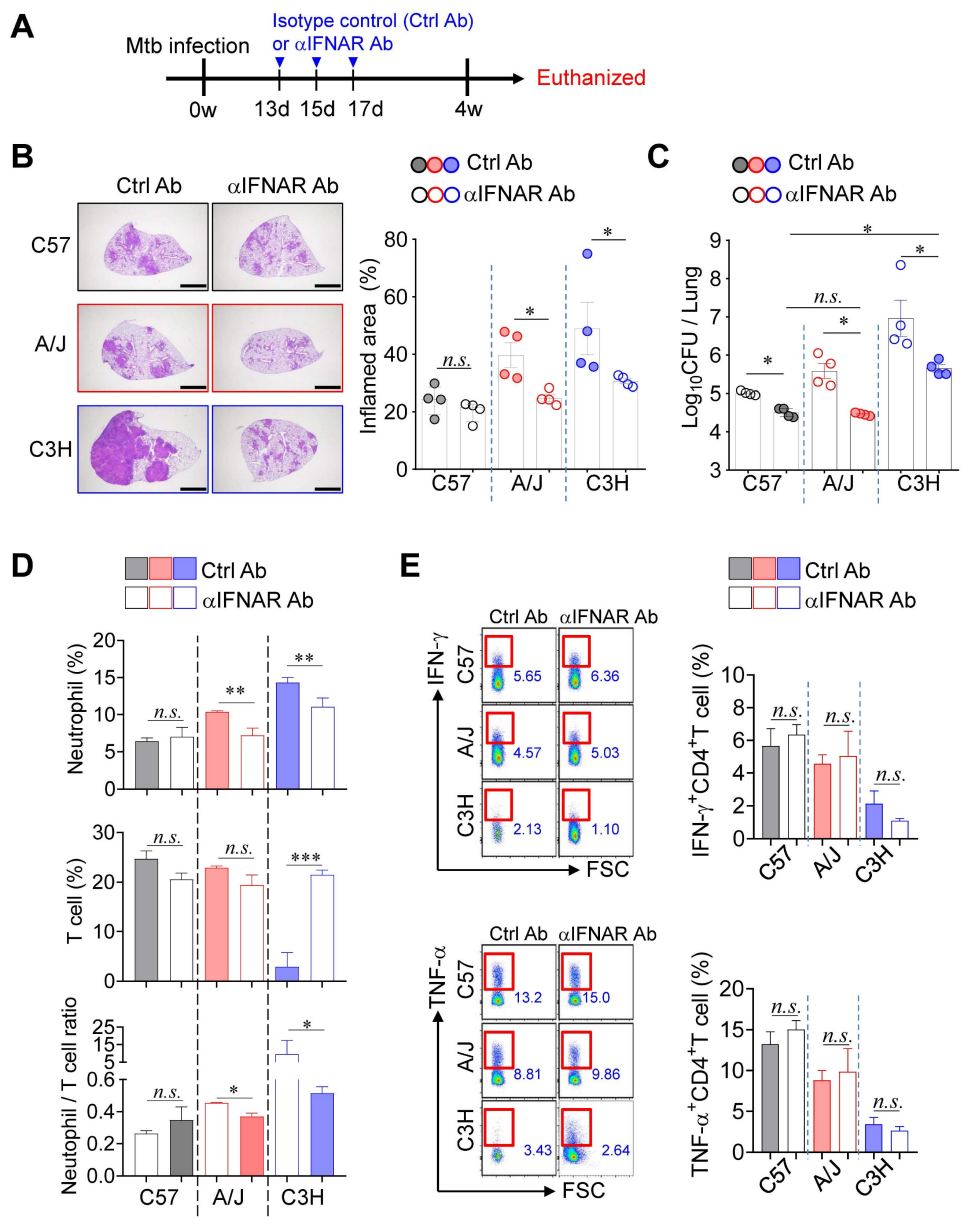
** Strain-dependent reduction of TB susceptibility by type I IFN signaling blockade.** (A) Schematic of experimental timeline. Mice were infected with Mtb K strain and treated with anti-IFNAR antibody at 13, 15, and 17 days post-infection to block type I IFN signaling. (B) Lung histopathology was analyzed using H&E staining (scale bar = 2 mm), and (C) bacterial burdens in the lungs at 4 weeks post-infection were assessed. *n* = 4. (D) Neutrophil and T cell in the lungs were analyzed by flow cytometry at 4 weeks post-infection. *n* = 4. (E) Lung cells were stimulated with ESAT-6 to analyze IFN-γ or TNF-α producing CD4^+^ T cells.* n* = 4. Data are presented as mean ± SD. Statistical analyses were performed using one-way ANOVA with Tukey's multiple comparison test. *n.s.* = not significant. *P < 0.05, **P < 0.01, ***P < 0.001.

Given that type I IFN signaling is a key pathogenic driver of TB exacerbation, particularly in association with increased neutrophil infiltration in both experimental and clinical settings 20, 24, we investigated the role of type I IFN signaling in TB immunopathology of TB-susceptible mouse strains. Notably, while no significant differences in pulmonary Mtb burden were observed at 2 weeks PI, a marked increase was evident at 4 weeks PI (Fig. 1). To further explore this transition, we assessed lung Mtb burden and *Ifnb* expression at 3 weeks PI, which revealed a concurrent increase in CFU and a significant upregulation of *Ifnb* expression (Fig. S6A-C). To determine the functional impact of type I IFN signaling, we administered a blocking anti-IFNAR antibody (αIFNAR Ab) and evaluated its effects in both TB-susceptible and TB-resistant mouse strains (Fig. 5A). Notably, αIFNAR Ab treatment significantly attenuated TB immunopathology, as evidenced by a substantial reduction in pulmonary bacterial burden and inflammation (Fig. 5B, C). Moreover, neutrophil frequencies in the lungs were markedly decreased in TB-susceptible mice, leading to a reduced N/T ratio. In the C3H/HeJ strain, a restoration of T cell frequency was also observed (Fig. 5D). Interestingly, despite the recovery in T cell numbers, antigen-specific T cell responses were not restored in either TB-susceptible or -resistant strains (Fig. 5E). Collectively, these findings suggest that attenuation of TB severity in TB-susceptible mice upon blockade of type I IFN signaling is associated with reduced neutrophil infiltration and a rebalanced N/T ratio, rather than full restoration of T cell function.

### IL-10 signaling blockade prevented TB immunopathology in A/J mouse strain but showed limited effect in C3H/HeJ strain

**Figure 6:**
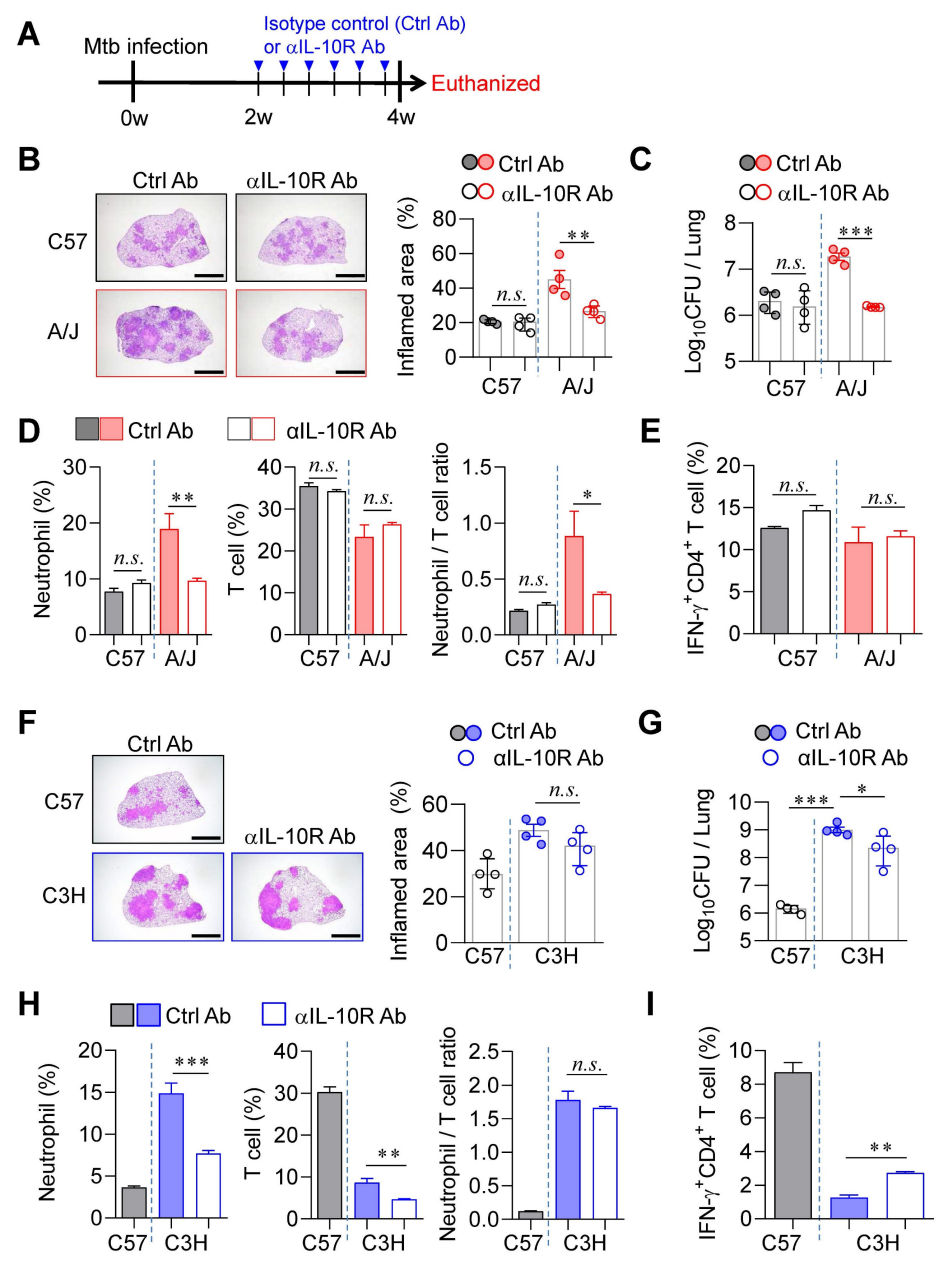
** Differential effects of IL-10 signaling blockade on TB susceptibility in A/J and C3H mice.** (A) Mice were infected with Mtb K strain and treated with anti-IL-10R antibody 3 times per week from 2 to 4 weeks post-infection to block IL-10 signaling. (B) Lung histopathology of C57BL/6 and A/J was analyzed using H&E staining (scale bar = 2 mm), and (C) bacterial burdens in the lungs at 4 weeks post-infection were assessed. *n* = 4. (D) Neutrophil and T cell in the lungs were analyzed by flow cytometry at 4 weeks post-infection. *n* = 4. (E) Lung cells were stimulated with ESAT-6 to analyze IFN-γ producing CD4^+^ T cells. *n* = 4. (F) Lung histopathology of C57BL/6 and C3H/HeJ was analyzed using H&E staining (scale bar = 2 mm), and (G) bacterial burdens in the lungs at 4 weeks post-infection were assessed. *n* = 4. (H) Neutrophil and T cell in the lungs were analyzed by flow cytometry at 4 weeks post-infection. (I) Lung cells were stimulated with ESAT-6 to analyze IFN-γ-producing CD4^+^ T cells. *n* = 4. Data are presented as mean ± SD. Statistical analyses were performed using one-way ANOVA with Tukey's multiple comparison test. *n.s.* = not significant. *P < 0.05, **P < 0.01, ***P < 0.001.

Previous studies have demonstrated that excessive type I IFN signaling induces the immunosuppressive cytokine IL-10, which exacerbates TB by suppressing IL-1α/β-mediated protective responses, particularly in a poly(I:C)-treated animal model 56. To determine whether IL-10 signaling contributes to TB progression in our model, we administered an anti-IL-10 receptor antibody (αIL-10R Ab) to both A/J and C3H/HeJ mice (Fig. 6A). In A/J mice, αIL-10R Ab treatment initiated at 2 weeks PI significantly ameliorated TB immunopathology. This was evidenced by a substantial reduction in pulmonary Mtb burden and associated inflammation (Fig. 6B, C), along with markedly decreased neutrophil frequencies and a lowered N/T ratio (Fig. 6D). However, antigen-specific T cell responses remained unaffected (Fig. 6E). In contrast, IL-10R blockade in C3H/HeJ mice resulted in only modest reductions in pulmonary Mtb burden and failed to reduce lung inflammation (Fig. 6F, G). Although neutrophil frequencies were significantly decreased, T cell frequencies were also reduced, resulting in no significant change in the N/T ratio (Fig. 6H). Interestingly, a slight increase in antigen-specific IFN-γ⁺ CD4⁺ T cell responses was observed (Fig. 6I). To further investigate the strain-specific effects of IL-10 signaling blockade, we examined GMPs in the BM of both strains at 4 weeks PI. This analysis was prompted by earlier findings of differential GMP increase levels in TB-susceptible versus TB-resistant strains (Fig. 4). Notably, IL-10R blockade led to a marked reduction in BM GMP increase ratio in A/J mice (Fig. S7A), whereas no significant changes were observed in C3H/HeJ mice (Fig. S7B), and this pattern was similar to the level of G-CSF in serum (Fig. S7C, D). These results suggest that the differential effects of IL-10 signaling blockade may be linked to distinct patterns of GMP expansion.

Taken together, while blockade of type I IFN signaling robustly attenuates TB progression in both susceptible strains (Fig. 5), IL-10 signaling blockade exerts a pronounced protective effect only in A/J mice. Its limited efficacy in C3H/HeJ mice underscores strain-specific differences that appear to be associated with changes in the N/T ratio in the lungs and GMP levels in the BM.

### Age-dependent mitigation on TB Severity was specific to A/J strain but limited to C3H/HeJ mice

**Figure 7:**
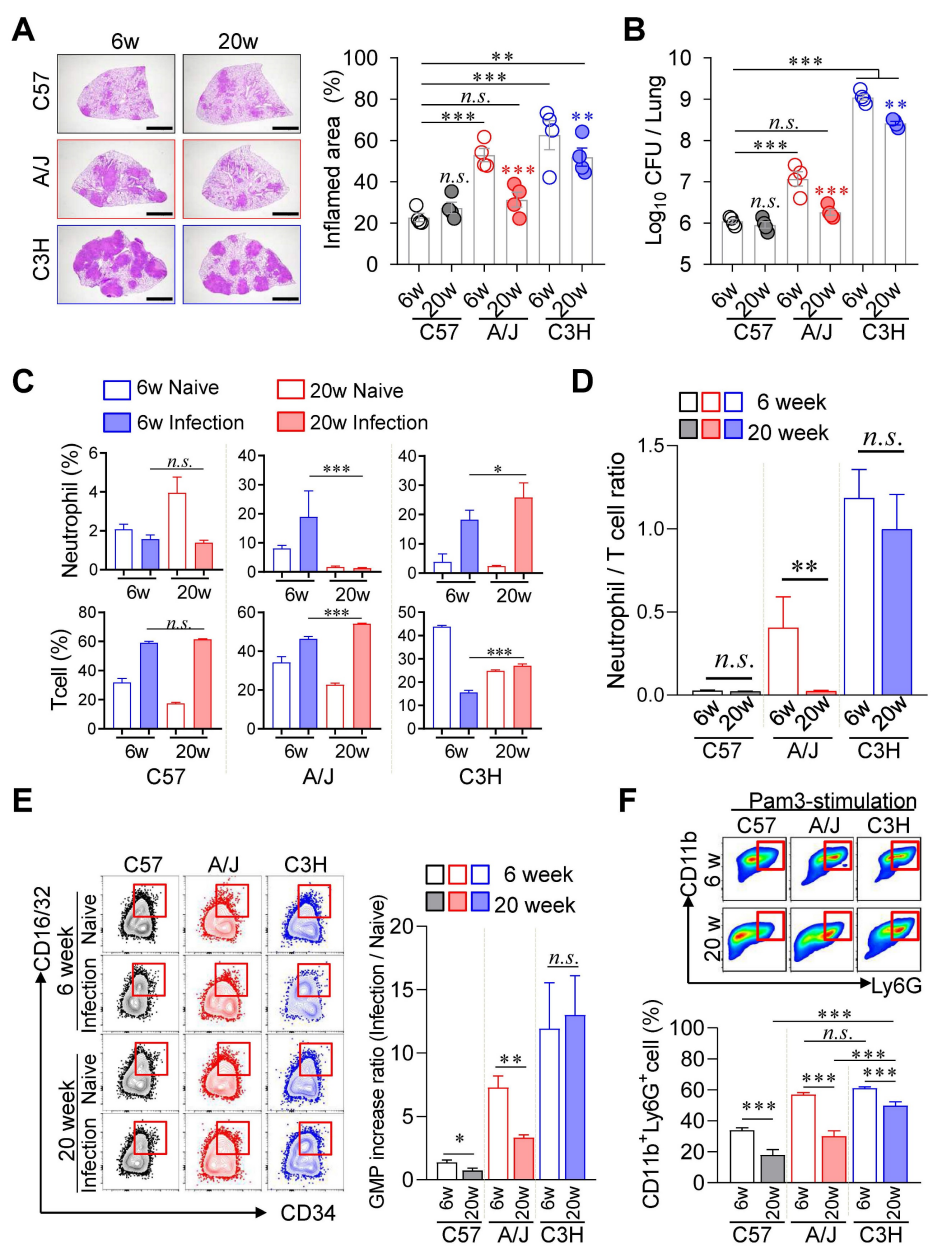
** Age-associated TB susceptibility linked to neutrophil-to-T cell ratio.** Six-week-old or twenty-week-old mice of the C57BL/6, A/J, and C3H/HeJ strains were challenged with Mtb. At 4 weeks post-infection, the mice were sacrificed. (A) Lung histopathology of 6-week-old and 20-week-old mice of each mouse strain was analyzed using H&E staining (scale bar = 2 mm), and (B) the lung bacterial burdens were assessed. *n* = 4. (C) Neutrophil and T cell in the lungs were analyzed by flow cytometry at 4 weeks post-infection. (D) Neutrophil-to-T cell ratio in the lungs at 4 weeks post-infection. *n* = 4. (E) The bone marrow cells were isolated, and the GMP population was analyzed by flow cytometry. *n* = 4. (F) The bone marrow cells were cultured with Pam3 in the presence of GM-CSF. After 6 days of culture, the CD11b^+^Ly6G^+^ population was analyzed by flow cytometry. *n* = 3. Data are presented as mean ± SD. Statistical analyses were performed using one-way ANOVA with Tukey's multiple comparison test. *n.s.* = not significant. *P < 0.05, **P < 0.01, ***P < 0.001. GMP, granulocyte-monocyte progenitor.

Given that younger children experience more severe TB disease than older children 57, we investigated whether age similarly influences TB pathogenesis in murine models. We compared disease progression in TB-susceptible (A/J and C3H/HeJ) and TB-resistant (C57BL/6J) mouse strains at 6 and 20 weeks of age. Notably, aging conferred a pronounced protective effect in A/J mice, with significantly reduced pulmonary TB pathology and Mtb burden in 20-week-old mice compared to their 6-week-old counterparts (Fig. 7A, B). In contrast, C3H/HeJ mice exhibited only modest age-associated improvement, while no significant age-related differences were observed in the resistant C57BL/6J strain. In A/J mice, aging was associated with reduced neutrophil infiltration and increased T cell frequencies in the lungs, resulting in a markedly lower N/T ratio (Fig. 7C, D). Conversely, aging in C3H/HeJ mice led to increases in both neutrophil and T cell frequencies, yielding no significant change in the N/T ratio. To explore the mechanistic basis of this age-dependent protection, we assessed GMP populations in the BM. Only A/J mice displayed a significant age-associated decline in the level of GMP increase ratio, we confirmed that this pattern correlated with G-CSF levels in serum (Fig. S8A). Compared to 6 weeks of age mice, G-CSF levels in serum of C3H/HeJ mice also decreased at 20 weeks of age, but remained at relatively high levels (Fig. S8A). These results suggest that reduced availability of myeloid progenitors may contribute to diminished inflammation and improved disease outcomes in older A/J mice (Fig. 7E).

We next evaluated the neutrophil differentiation potential of BM cells under inflammatory conditions. Upon stimulation with GM-CSF and Pam3, BM cells from 20-week-old A/J mice generated over 50 % fewer CD11c⁻CD11b⁺Ly6G⁺ neutrophils compared to 6-week-old mice. In contrast, only a ~10 % reduction in neutrophil differentiation was observed in C3H/HeJ mice of the same age groups (Fig. 7F). Similar trends were observed following stimulation with ODNs (Fig. S8B, C).

Finally, we investigated the protective effects of Bacillus Calmette-Guérin (BCG) vaccination in both TB-susceptible strains, assessed 12 weeks after subcutaneous BCG administration. In A/J mice, which already exhibited attenuated TB pathology at 20-week-old mice, 18-week-old BCG-vaccinated mice showed complete protection without pulmonary immunopathology (Fig. S9A, B). Interestingly, this protection was associated with a significant reduction in T cell frequencies but not neutrophils, resulting in an increased N/T ratio (Fig. S9C). GMP increase levels in the BM were also significantly decreased following BCG vaccination (Fig. S9D). G-CSF levels in serum were not significantly different regardless of BCG vaccination (Fig. S9E). In contrast, 18-week-old C3H/HeJ mice still exhibited substantial TB immunopathology.

However, BCG vaccination markedly protected against lung inflammation and reduced Mtb burden (Fig. S9F, G). This protective effect was accompanied by a significant decrease in neutrophil frequencies and an increase in T cell frequencies, leading to a pronounced reduction in the N/T ratio (Fig. S9H). BCG vaccination also significantly reduced GMP proportions in the BM of C3H/HeJ mice (Fig. S9I). G-CSF levels in serum were significantly down-regulated in C3H/HeJ mice with BCG vaccination (Fig. S9J). Collectively, these findings suggest that age-associated protection in A/J mice is driven by reduced GMP levels and impaired neutrophil differentiation in response to inflammation, resulting in lower TB susceptibility in older animals. In contrast, the C3H/HeJ strain, which lacks substantial age-related protection, benefits significantly from BCG vaccination through reductions in both the N/T ratio and GMP levels, highlighting distinct mechanisms of protection in TB susceptible inbred mouse strains.

## Discussion

In this study, we investigated host-pathogen interactions in murine models using the highly virulent Mtb K strain to identify both common and strain-specific mechanisms underlying TB immunopathology. We employed two inbred mouse strains, C3H/HeJ and A/J, which exhibit distinct immune response patterns but share a common susceptibility to Mtb infection. Our findings reveal that neutrophilic inflammation, type I IFN signaling, and enhanced granulopoiesis converge as central drivers of disease progression in these models. Importantly, we identified the N/T ratio in the lungs and expansion of GMPs in the BM as key immunological determinants of TB severity. These features offer potential host-directed therapeutic targets for mitigating TB-related immunopathology.

TB severity is governed by complex interactions between host immune responses and bacterial virulence. Numerous factors influence the disease spectrum and progression of TB. Murine models have been instrumental in elucidating pathogenic mechanisms and evaluating therapeutic strategies. However, their translational relevance depends heavily on appropriate model selection and rigorous experimental design 58, 59. The diverse outbred mouse model exhibits variations in immune responses and disease outcomes driven by genetic heterogeneity, making it a valuable tool for reflecting the spectrum of human TB disease 60. Similarly, inbred mouse strains differ significantly in their immune response profiles 61, reflecting variations in cellular composition and immune architecture between mice and humans 62. Therefore, it is essential to identify and validate conserved pathogenic mechanisms across models to ensure findings are clinically meaningful. Toward this goal, we focused on experimentally and clinically established factors, type I IFN signature and neutrophils, as common mediators of TB immunopathogenesis 20, 24, 51, 63.

Despite exhibiting minimal lung pathology and comparable bacterial burdens during early infection, both C3H/HeJ and A/J mice progressed rapidly to severe disease at 4 weeks PI. This disease progression was characterized by intense pulmonary inflammation, necrotic lesions, and markedly increased Mtb loads. Importantly, this was accompanied by pronounced neutrophilic infiltration and elevated expression of *Ifnb*. Functional studies confirmed the pathogenic roles of both neutrophils and type I IFN signaling: antibody-mediated neutrophil depletion and blockade of the type I IFN receptor significantly ameliorated disease severity in both strains. These findings implicate neutrophil accumulation and type I IFN signaling as common pathogenic mechanisms in these TB-susceptible models and extend to other susceptible mouse strains.

Consistent immunopathological features have been reported in additional TB-susceptible models. In ATG5 (autophagy-related gene 5) KO mice 64, C3HeB/FeJ mice 22, and 129S2 mice 65, elevated type I IFN responses and heightened neutrophilic inflammation have been implicated in exacerbated disease. Notably, in both ATG5 KO and C3HeB/FeJ mice, type I IFN promotes the formation of NETs, which support bacterial persistence and worsen disease outcomes. In addition, studies of *Sp140^-/-^* mice showed that excessive DNA-rich NETs activate plasmacytoid dendritic cells, driving a feed-forward loop of elevated type I IFN production, which results in impaired IFN-γ production and increased Mtb growth 20, 24, 51, 63. Similarly, in a viral co-infection model using C57BL/6J mice, type I IFN activity suppresses Mtb-specific IFN-γ responses, leading to increased bacterial burden and exacerbated pulmonary inflammation. Blockade of type I IFN in this setting restores protective Th1 responses, reduces neutrophil accumulation, and improves lung pathology 21. In our study, we used C3H/HeJ mice. Unlike C3HeB/FeJ mice, which have high TB susceptibility linked to the "super susceptibility to tuberculosis-1" (*sst1*) locus on chromosome 1 66, C3H/HeJ mice have been reported to harbor a loss-of-function mutation in TLR4 25. However, C3H/HeJ mice still exhibit high TB susceptibility, and we confirmed type I IFN-mediated disease exacerbation, highlighting a mechanism by which early type I IFN signaling interferes with the development of protective immunity. On the other hand, although type I IFN signaling does not directly impair T cell responses in 129S2 mice, it alters neutrophil recruitment through CXCL1/CXCL5-mediated chemotaxis, resulting in alveolar infiltration and inflammation, phenocopying the immunological response observed in A/J mice 65. Moreover, Saqib et al. showed in *Il1r1^-/-^* C57BL/6J mice that type I IFN suppresses neutrophil bacterial uptake while driving influx of immature CD101⁻ neutrophils, which promote bacterial persistence, epithelial injury, and reduced surfactant production 51. In line with these reports, we found that increased type I IFN, ROS, and NET formation, along with decreased NOS2, were associated with a high Mtb burden in neutrophils of susceptible mice. Overall, the convergence of these features across diverse TB-susceptible mouse models as well as human TB 24, 63 underscores the central role of type I IFN-mediated neutrophilic inflammation as a key driver of TB immunopathogenesis.

An intriguing finding in this study is the differential effect of IL-10 signaling blockade between the two TB-susceptible strains. While inhibition of type I IFN signaling robustly attenuated TB progression in both A/J and C3H/HeJ mice, IL-10 receptor blockade conferred significant protection only in A/J mice. The limited therapeutic effect in C3H/HeJ mice suggests strain-specific differences in IL-10-mediated immunopathology. Mechanistically, type I IFN is known to promote IL-10 production in Mtb-infected macrophages by stabilizing *Il10* mRNA, leading to suppression of proinflammatory cytokines such as TNF-α, IL-12, and IL-1β, and impairing IFN-γ-induced macrophage activation and bacterial killing 67. Furthermore, in a model of influenza A virus co-infection, type I IFN-driven IL-10 production impaired the initiation and expansion of Mtb-specific CD4⁺ T cell responses. In this context, blockade of IL-10 signaling restored bacterial control to levels observed in Mtb-only infected mice 68. These data suggest that the deleterious role of IL-10 signaling primarily affects the adaptive T cell response. Consequently, in C3H/HeJ mice, where CD4⁺ T cell responses are already profoundly compromised, IL-10 signaling blockade offers limited benefit, reinforcing the idea that the effectiveness of such intervention depends on the presence of functional T cell immunity.

The most notable finding in this study is the divergence in TB disease severity between A/J and C3H/HeJ mice, despite both exhibiting common pathogenic features such as excessive pulmonary neutrophilic infiltration and heightened type I IFN signaling. This disparity is likely attributable to differences in the quality and magnitude of adaptive immune responses. A/J mice retained pulmonary T cell populations and mounted robust antigen-specific IFN-γ⁺ CD4⁺ T cell responses, whereas C3H/HeJ mice showed a marked reduction in T cell frequencies and negligible antigen-specific responses. The absence of effective T cell immunity in C3H/HeJ mice was associated with more severe neutrophilic inflammation and exacerbated TB pathology. These findings align with prior studies indicating that failure to recruit protective IFN-γ-producing CD4⁺ T cells contributes to heightened susceptibility in mouse models with excessive type I IFN responses 21, 22. Moreover, IFN-γ has been shown to suppress neutrophil accumulation and promote neutrophil apoptosis, suggesting a protective role in limiting neutrophil-mediated tissue damage 40. Interestingly, in A/J mice, antigen-specific T cell responses were not significantly improved even after TB pathology was remarkably attenuated through neutrophil depletion or type I IFN signaling blockade. This indicates that these interventions primarily modulate the innate immune compartment without impairing T cell function. A recent study by Gern et al. demonstrated that preexisting CD4⁺ T cells protect against neutrophil-driven necrotic pathology in C3HeB/FeJ mice 69, highlighting the critical role of T cell abundance in restraining neutrophil-mediated damage. Consistently, Zelmer et al. emphasized that the quantity of T cells, rather than the magnitude of antigen-specific IFN-γ production, is the key determinant of BCG-mediated protection in TB-susceptible strains 70.

Among all evaluated parameters in this study, the pulmonary N/T ratio emerged as the most reliable correlate of TB immunopathology severity across strains and experimental conditions. Notably, interventions that attenuated disease, including neutrophil depletion, type I IFN, IL-10 signaling blockade, BCG vaccination, and age-related modulation, were all associated with reduced N/T ratios and improved lung pathology in both TB-susceptible mice. These findings support the translational relevance of the N/T ratio as a biomarker for TB severity. In clinical settings, the peripheral blood N/L ratio has shown diagnostic utility in distinguishing TB from bacterial pneumonia 71 and is strongly associated with increased disease risk and poor outcomes in severe TB forms, such as miliary TB, cavitary TB, and TB meningitis 34, 71-74. Furthermore, Panteleev et al. reported that clinical TB severity correlated more strongly with the N/T ratio than with Mtb-specific cytokine-producing CD4⁺ T cell response 75, suggesting that shifts in neutrophil-T cell balance may serve as a key determinant of TB progression. These findings underscore the potential of targeting the neutrophil-T cell axis and restoring T cell numbers as promising strategies to mitigate TB immunopathology. In parallel with the strong inverse correlation between the pulmonary N/T ratio and TB severity observed in this study, we also found that increased ratios of GMPs in the BM following Mtb infection and immunological interventions closely mirrored TB immunopathology in both TB-susceptible strains. While GM-CSF typically promotes dendritic cell differentiation over granulocytes *in vitro* 76, our data indicate that BM cells from TB-susceptible mice shift toward neutrophil-like differentiation under TLR agonists-induced inflammatory conditions.

Recent findings support these observations. Bobba et al. reported that Mtb strains that induce high levels of type I IFN skew granulopoiesis *in vivo* 77. Although literature on granulopoiesis in A/J mice is limited, studies in mice with a C3H background show that type I IFN drives granulopoiesis 20, and that these mice develop progressive neutrophilia under chronic inflammatory conditions, such as tumor development 78. Recently, Li et al. demonstrated through bone marrow cell sequencing that chronic local inflammation can drive systemic inflammation by promoting neutrophil-lineage commitment of hematopoietic stem and progenitor cells via type I IFN-induced transcription program 79. Our previous work demonstrated that G-CSF is associated with GMP expansion and neutrophil accumulation during exacerbated Mtb immunopathology 80. Furthermore, the administration of purified human native G-CSF to C3H/HeJ, but not C57BL/6N, mice robustly accelerated granulopoiesis and significantly elevated peripheral neutrophil counts 81. More recently, it was reported that both G-CSF and CXCR2 are critical for the accumulation of pathogenic neutrophils in the lungs under type I IFN-driven TB immunopathology 51. In accordance with previous reports, we observed that circulating G-CSF levels in both TB-susceptible strains positively correlated with GMP frequencies in the BM, pulmonary neutrophil infiltration, and overall TB disease severity, both during infection and following immunomodulatory interventions. Collectively, these results suggest that heightened G-CSF signaling may promote granulopoiesis during type I IFN-driven or TB-associated immunopathology.

Importantly, BCG vaccination significantly attenuated the expansion of GMPs in the BM of both TB-susceptible strains. Previous studies have shown that while BCG reprograms HSCs toward balanced myelopoiesis, virulent Mtb reprograms HSCs via a type I IFN-dependent mechanism that skews differentiation away from protective myeloid lineages 82, 83. This suggests that either the BCG vaccine itself 40 or preexisting Mtb-specific IFN-γ-producing T cells 83-85 may restrain pathological granulopoiesis by maintaining appropriate HSC differentiation programs in TB-susceptible mice. Together, these findings highlight the potential of targeting BM hematopoietic programming, particularly the G-CSF/GMP axis, as a strategy to mitigate neutrophil-driven TB immunopathology.

Our study has several limitations that warrant consideration. First, despite the use of two TB-susceptible inbred mouse strains, their genetic backgrounds remain limited in reflecting the genetic heterogeneity of human populations. Future studies using outbred or genetically heterogeneous mouse panels would be valuable to validate the broader applicability of the pulmonary N/T ratio and BM GMP dynamics as pathogenic grounds of TB severity. In support of the clinical relevance of these parameters, previous studies reported that peripheral blood from TB patients shows a significantly higher N/L ratio 86, 87, and increased G-CSF level than that of healthy individuals, with this elevation correlating positively with clinical disease severity indicators 88. Moreover, whole-blood transcriptomic analyses from human TB cohorts have revealed widespread upregulation of neutrophil-associated gene modules alongside diminished T cell effector signatures 23, suggesting that evaluation of these indicators in clinical cohorts would be valuable. Second, although aging conferred substantial protection in A/J mice, evidenced by reduced lung pathology, lower neutrophil frequencies, and diminished GMP populations in the BM, the underlying mechanisms of age-dependent protection in this strain remain to be elucidated. One possible explanation for the enhanced protection observed in 20-week-old A/J mice is age-dependent hematopoietic remodeling, which may contribute to reduced granulopoiesis and neutrophil accumulation during Mtb infection. Hematopoiesis has been reported to be affected by aging in both human and mouse models 89, 90, and in particular, the age-related decrease in G-CSF responsiveness 91, 92 suggests that aging may modulate neutrophil generation through reduced granulopoiesis. Elucidating how aging influences the regulation of granulocyte and neutrophil production during TB progression using a physiologically relevant age model will deepen our understanding of age-related differences in TB susceptibility.

Despite these limitations, our study identifies reliable immunological correlates of TB severity that are robust across genetically distinct models and experimental interventions. This underscores the potential utility of these inbred strains as powerful and complementary tools for modeling TB progression and dissecting the immunopathological mechanisms underlying host susceptibility.

Taken together, our findings provide compelling evidence that two genetically distinct TB-susceptible mouse strains, C3H/HeJ and A/J, develop severe TB immunopathology through distinct but converging mechanisms characterized by heightened type I IFN signaling and excessive neutrophilic inflammation. Our study advances current understanding of TB pathogenesis by identifying the pulmonary N/T ratio and BM GMP dynamics as key determinants of disease severity. These immunological metrics not only yield mechanistic insights into neutrophil-driven TB immunopathology but also position C3H/HeJ and A/J mice as robust, complementary platforms for investigating the spectrum of TB progression and evaluating host-directed therapies.

## Supplementary Material

Supplementary figures.

## Data Availability

All data relevant to the study are included in the article or uploaded as supplementary information.
