# Supplementary material for: Exploring Host-driven Immunopathological Factors Developing Severe Tuberculosis: Insights from Comparative Mouse Models
Source: Int J Biol Sci. 2026 Feb 4;22(5):2512–32. doi: 10.7150/ijbs.124878 (PMC12965220; doi:10.7150/ijbs.124878)
Supplement: Supplementary file 1 — Supplementary figures. [file ijbsv22p2512s1.pdf]

Supplementary materials

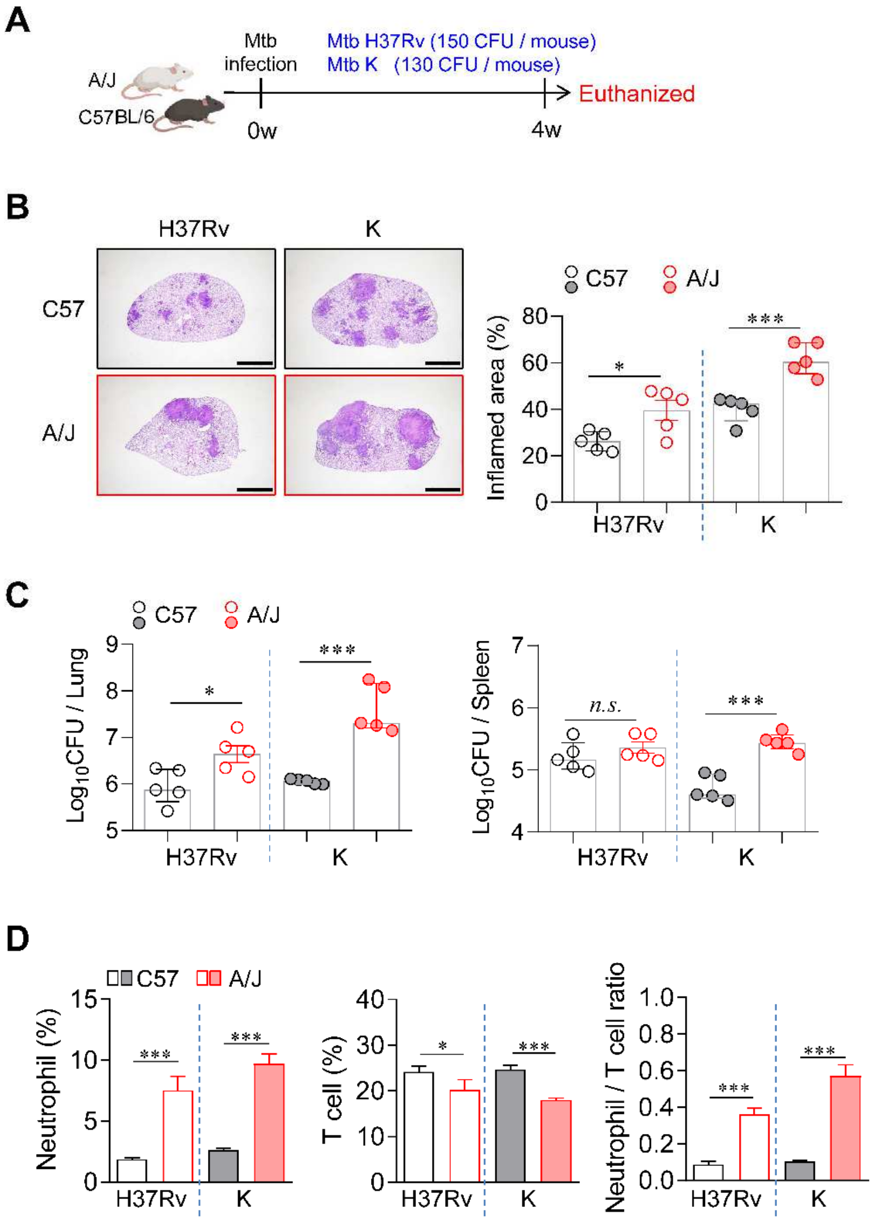

Figure S1. Comparative evaluation of different virulence between Mtb K strain and H37Rv in TB-susceptible A/J mice. (A) C57BL/6 and A/J mice were challenged with Mtb H37Rv (~150 CFU/mouse) or K strain (~130 CFU/mouse), and sacrificed at 4 weeks post-infection. (B) Lung histopathology was analyzed using H&E staining (scale bar = 2 mm), and (C) bacterial burdens in the lung and spleen at 4 weeks post-infection were assessed. $n = 5$. (D) Neutrophil and T cell in the lungs were analyzed by flow cytometry at 4 weeks post-infection. $n = 5$. Data are presented as mean ± SD. Statistical analysis was performed by one-way ANOVA with Tukey's multiple comparisons. *n.s.* = not significant, $*p < 0.05$, and $***p < 0.001$.

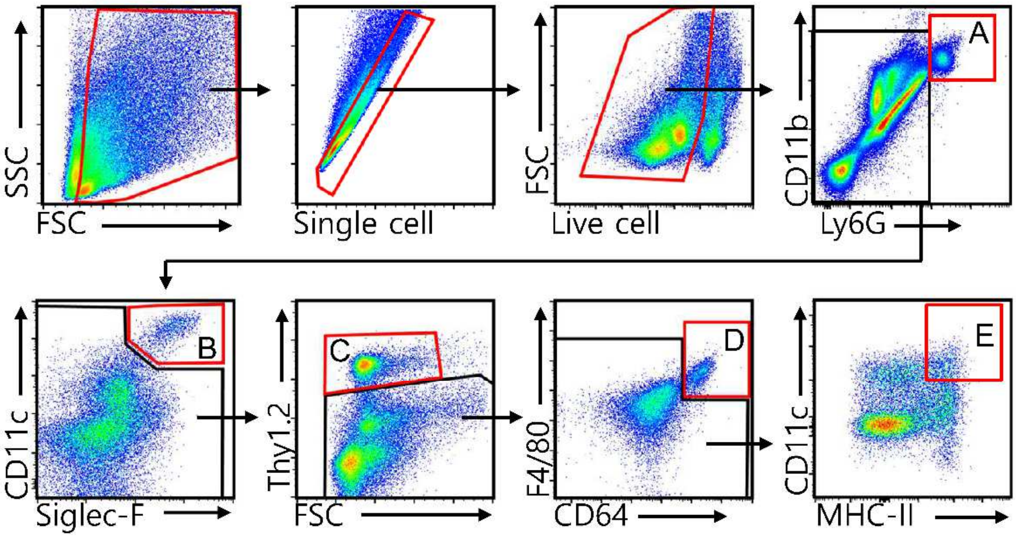

A: Neutrophil
B: Alveolar macrophage
C: T cell
D: Interstitial macrophage
E: Dendritic cell

Figure S2. Sequential gating strategies for lung immune cell populations. Lung cell suspensions were analyzed by flow cytometry to determine the immune cell populations. Firstly, an inclusion gate was drawn around cells with equivalent forward scatter-height and forward scatter-area values to exclude doublets and larger cell aggregates. Live cells were gated on LIVE/DEAD viability dye-negative cells. $CD11b^{+}Ly6G^{+}$ and $CD11c^{+}Siglec\text{-}F^{+}$ populations were excluded sequentially. T cells were gated on the $Thy1.2^{+}$ population, and $CD64^{+}F4/80^{+}$ cells were excluded from $Thy1.2^{-}$ cells. DCs were gated on $CD11c^{+}MHC\text{-}II^{+}$ population.

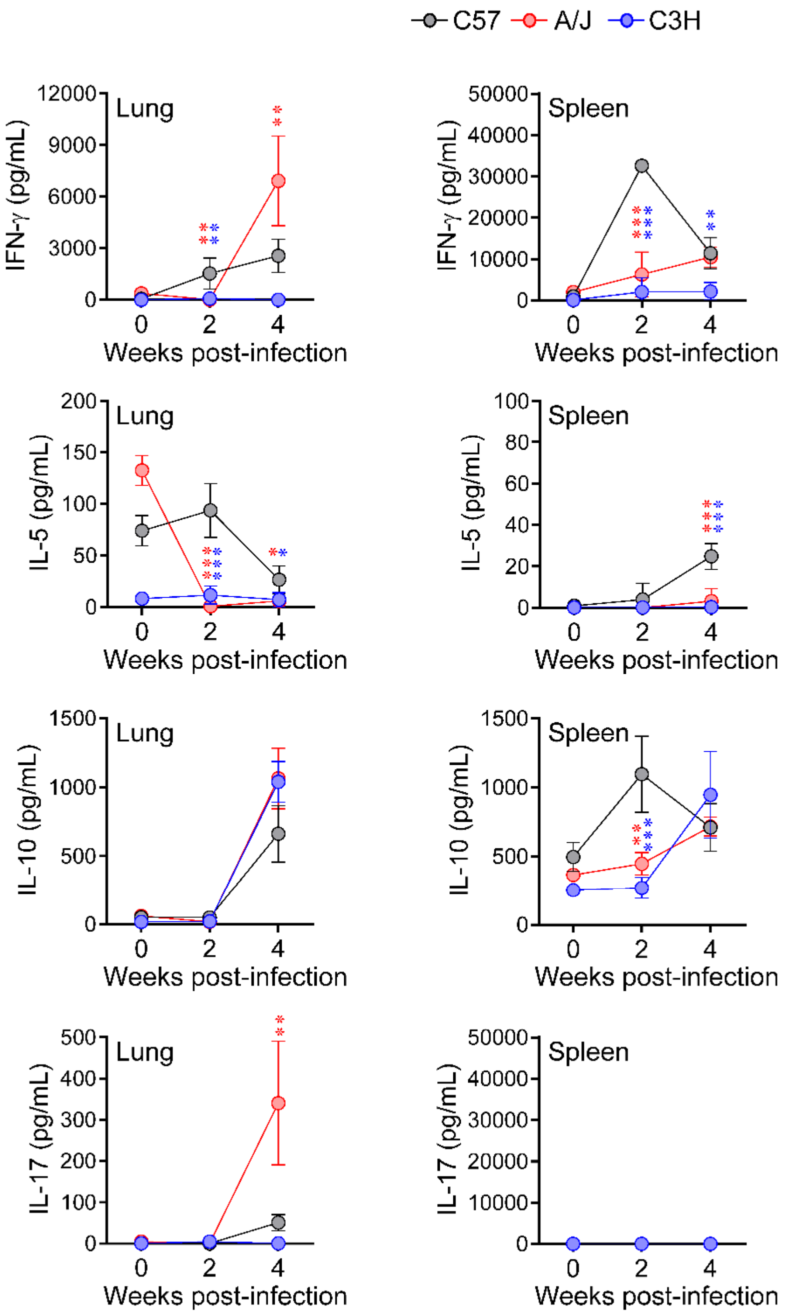

Figure S3. Differential antigen-specific cytokine profiles among different mouse strains during Mtb infection. Lung and spleen cells from indicated time points were stimulated with ESAT-6 for 12 hours, and the levels of cytokines were analyzed with ELISA. Red and blue asterisks indicate comparisons of A/J vs. C57BL/6 and C3H/HeJ vs. C57BL/6 at the same time point, respectively. $n = 4$. Data are presented as mean ± SD. Statistical analysis was performed by one-way ANOVA with Tukey's multiple comparisons. *$p < 0.05$, **$p < 0.01$, and ***$p < 0.001$.

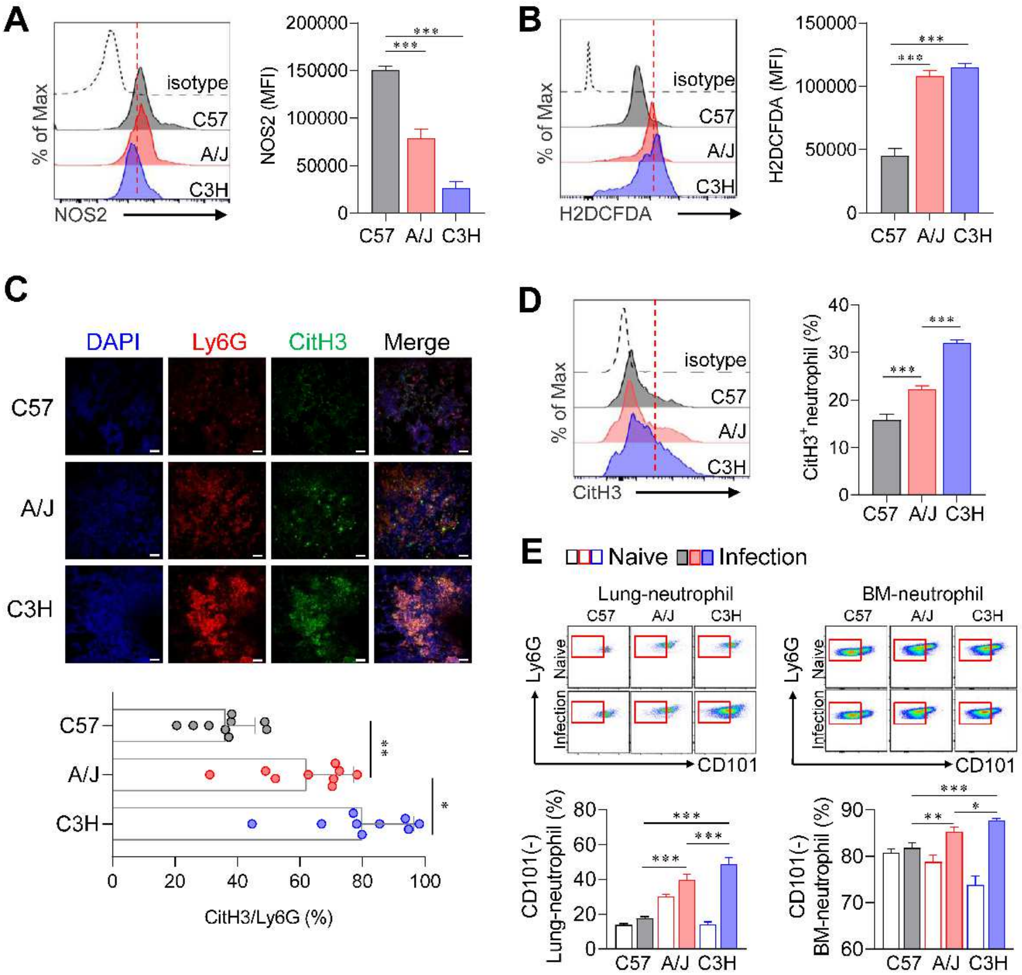

Figure S4. Functional and phenotypic divergence of neutrophils across mouse strains with differential susceptibility to Mtb infection. Lung neutrophils from C57BL/6, A/J, and C3H/HeJ mice were analyzed 4 weeks post-infection with Mtb K strain. The expression levels of NOS2 (A) and H2DCFDA (B) in lung neutrophils were measured by flow cytometry. $n = 4$. (C) Lung tissues were stained for Ly6G (red), CitH3 (green), and nuclei counterstained using DAPI (blue). Representative images of lung tissue were acquired from selected fields (scale bars, 100 µm). Quantification was performed using three mice per group ($n = 3$), with three randomly selected fields analyzed per mouse. NET area in the lung was expressed as a percentage normalized to the Ly6G-positive signal. (D) The CitH3^{+}neutrophils were measured with flow cytometry. $n = 4$. (E) CD101^{-} neutrophil subsets were analyzed in the lung and bone marrow. $n = 4$. Data are presented as mean ± SD. Statistical analyses were performed using one-way ANOVA with Tukey's multiple comparison test. *n.s.* = not significant. *$p < 0.05$, **$p < 0.01$, ***$p < 0.001$. NOS2, nitric oxide synthase 2; 2′,7′-dichlorodihydrofluorescein diacetate, H2DCFDA; CitH3, citrullinated histone H3; NET, neutrophil extracellular trap.

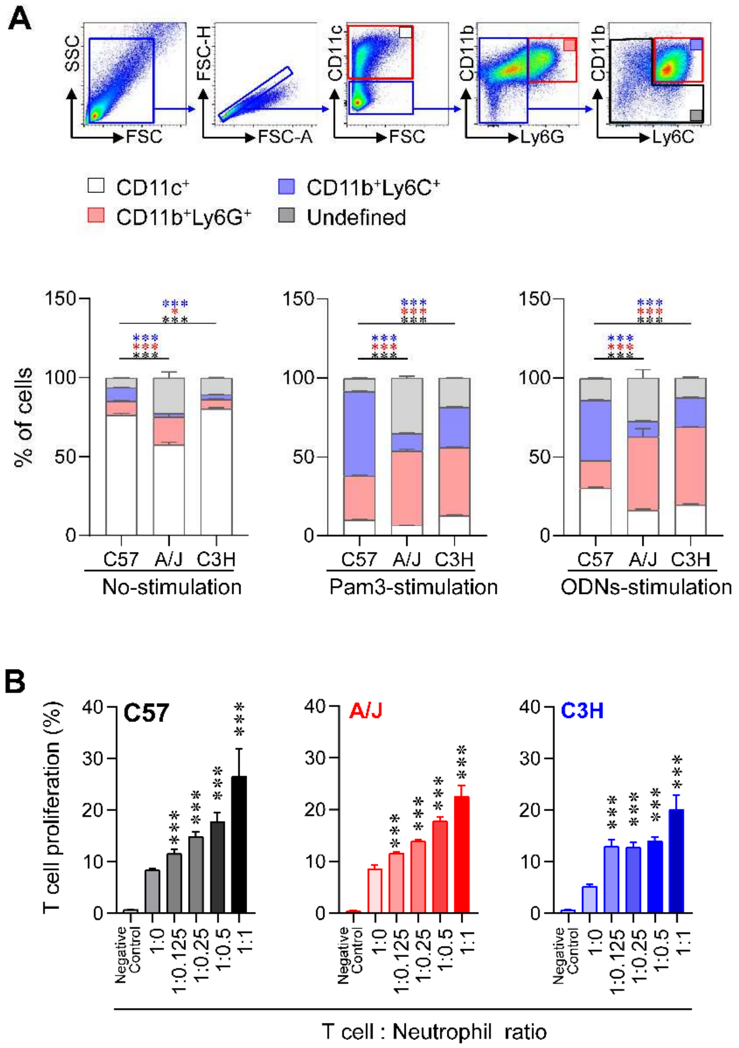

Figure S5. Phenotypic and functional characterization of pulmonary CD11b$^+$Ly6G$^+$ neutrophils and frequency and differentiation features bone marrow cells from Mtb-susceptible and -resistant mouse strains. (A) The bone marrow cells were cultured with Pam3 or ODNs in presence of GM-CSF. After 6 days of culture, CD11c$^+$, CD11c$^-$CD11b$^+$Ly6G$^+$, CD11c$^-$CD11b$^+$Ly6C$^+$ population were analyzed by flow cytometry. Black, red, and blue asterisks indicate group comparisons of CD11c$^+$, CD11c$^-$CD11b$^+$Ly6G$^+$, and CD11c$^-$CD11b$^+$Ly6C$^+$ populations, respectively. n = 3. (**B**) T cells were isolated from spleens of uninfected C57BL/6, A/J, and C3H/HeJ mice. The T cells were co-cultured with sorted lung CD11b$^+$Ly6G$^+$ cells from Mtb-infected C57BL/6, A/J, and C3H/HeJ mice at 4 weeks post-infection. n = 3. Undesignated asterisks indicate statistical comparisons relative to the T cell:neutrophil ratio of 1:0. Data are presented as mean ± SD. Statistical analyses were performed using one-way ANOVA with Tukey's multiple comparison test. *$p < 0.05$, ***$p < 0.001$.

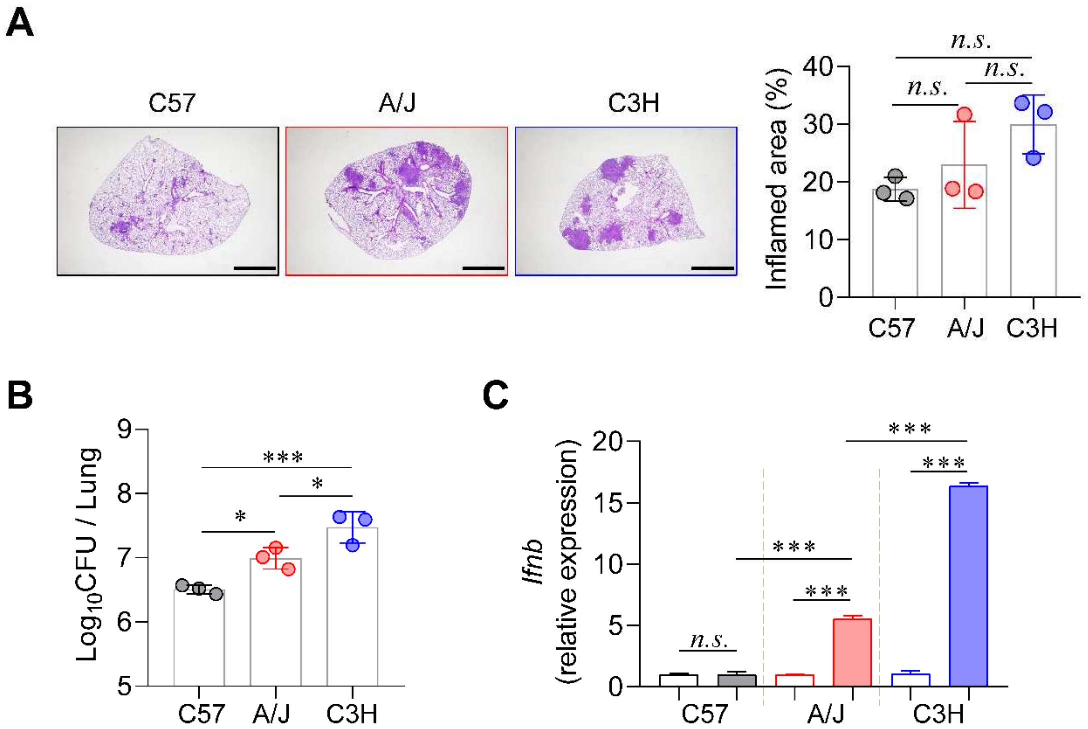

Figure S6. Pulmonary levels of *Ifnb* expression at 3 weeks post-infection in TB-susceptible and -resistant mouse strains. (A) Lung histopathology of inbred mouse strains (C57BL/6, A/J, and C3H/HeJ) was analyzed using H&E staining (scale bar = 2 mm), and (B) bacterial burdens in the lungs at 3 weeks post-infection were assessed. n = 3. (C) The expression level of *Ifnb* in lung tissues was measured by RT-PCR in C57BL/6, A/J, and C3H/HeJ mice at 3 weeks post-infection. n = 3. Data are presented as mean ± SD. Statistical analyses were performed using one-way ANOVA with Tukey's multiple comparison test. *n.s.* = not significant. *$p < 0.05$, ***$p < 0.001$.

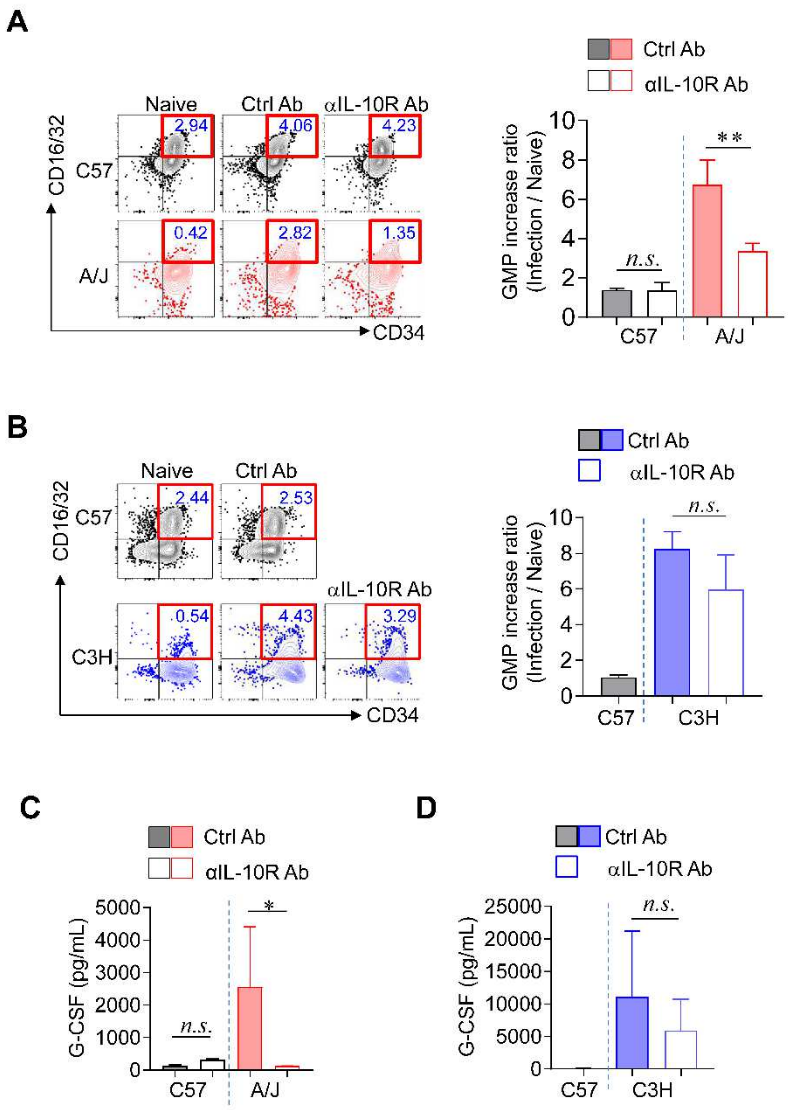

Figure S7. Differential effect of IL-10 signaling blockade–induced on granulocyte-monocyte progenitor increase ratio between A/J and C3H/HeJ mice. Mice were infected with Mtb K strain and treated with anti-IL-10R antibody 3 times per week from 2 to 4 weeks post-infection to block IL-10 signaling. (A) At 4 weeks post-infection, the bone marrow cells were isolated from C57BL/6, A/J with or without anti-IL-10R antibody treatment. The GMPs population were analyzed by flow cytometry. *n* = 3. (B) At 4 weeks post-infection, the bone marrow cells were isolated from C57BL/6, C3H/HeJ with or without anti-IL-10R antibody treatment. The GMP population was analyzed by flow cytometry. *n* = 3. At 4 weeks post-infection the G-CSF level in serum of C57BL/6, A/J (C), and C57BL/6, C3H/HeJ (D) with or without anti-IL-10R antibody treatment were measured by ELISA. *n* = 4. Data are presented as mean ± SD. Statistical analyses were performed using one-way ANOVA with Tukey's multiple comparison test. *n.s.* = not significant. $**p < 0.01$. GMP, granulocyte-monocyte progenitor.

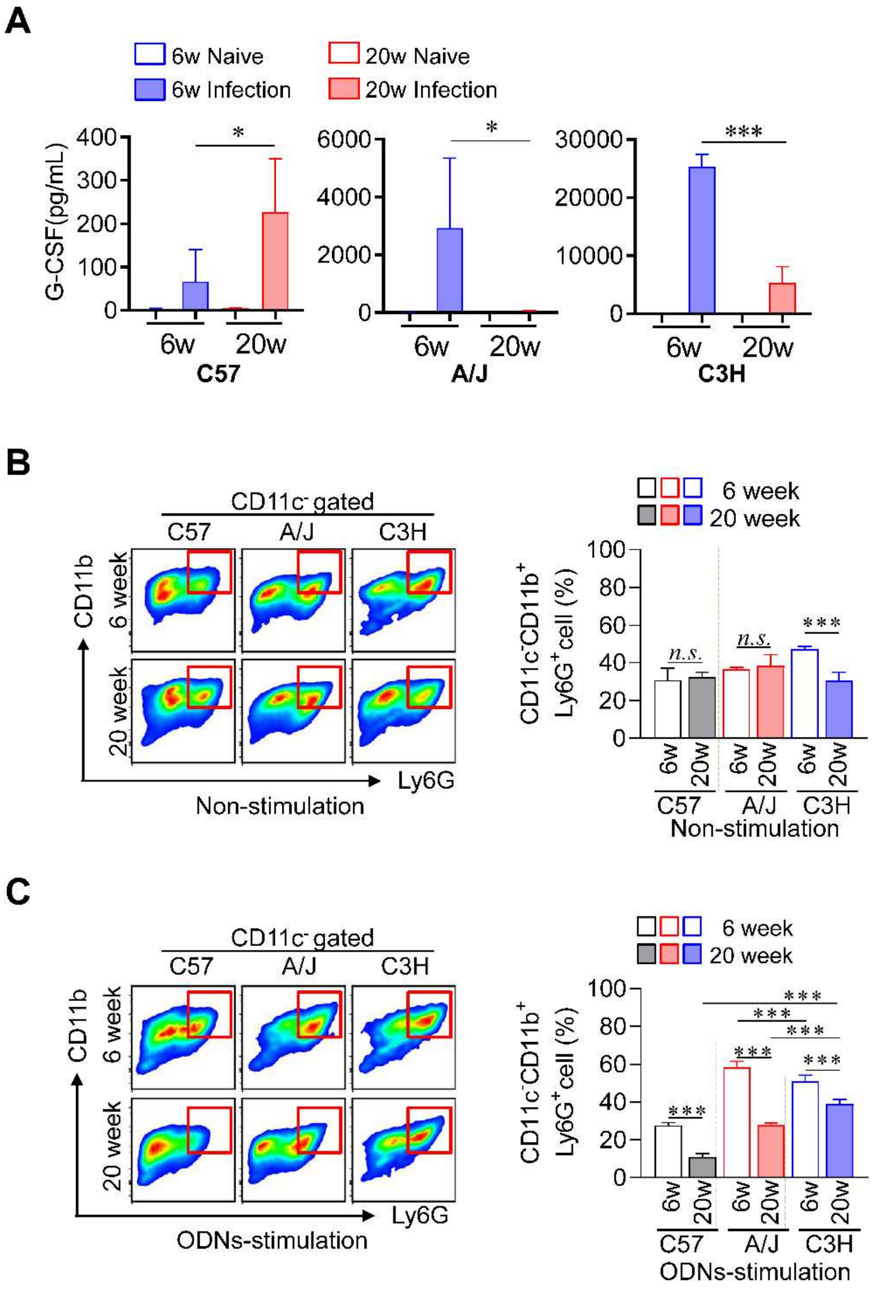

Figure S8. Differential age-dependent effect on granulocyte differentiation in naïve and TLR-induced inflammation conditions between A/J and C3H/HeJ mice upon GM-CSF treatment. Following up on the findings in Figure 7, (A) the G-CSF levels in serum of Mtb-infected C57BL/6, A/J, and C3H/HeJ were measured by ELISA. $n = 4$. The bone marrow cells were isolated from uninfected C57BL/6, A/J, and C3H/HeJ mice at 4 weeks post-infection and cultured (B) without or (C) with ODNs stimulation in the presence of GM-CSF. After 6 days of culture, $CD11b^{+}Ly6G^{+}$ populations were analyzed by flow cytometry. $n = 3$. Data are presented as mean ± SD. Statistical analyses were performed using one-way ANOVA with Tukey's multiple comparison test. *n.s.* = not significant. $*p < 0.05$, $***p < 0.001$.

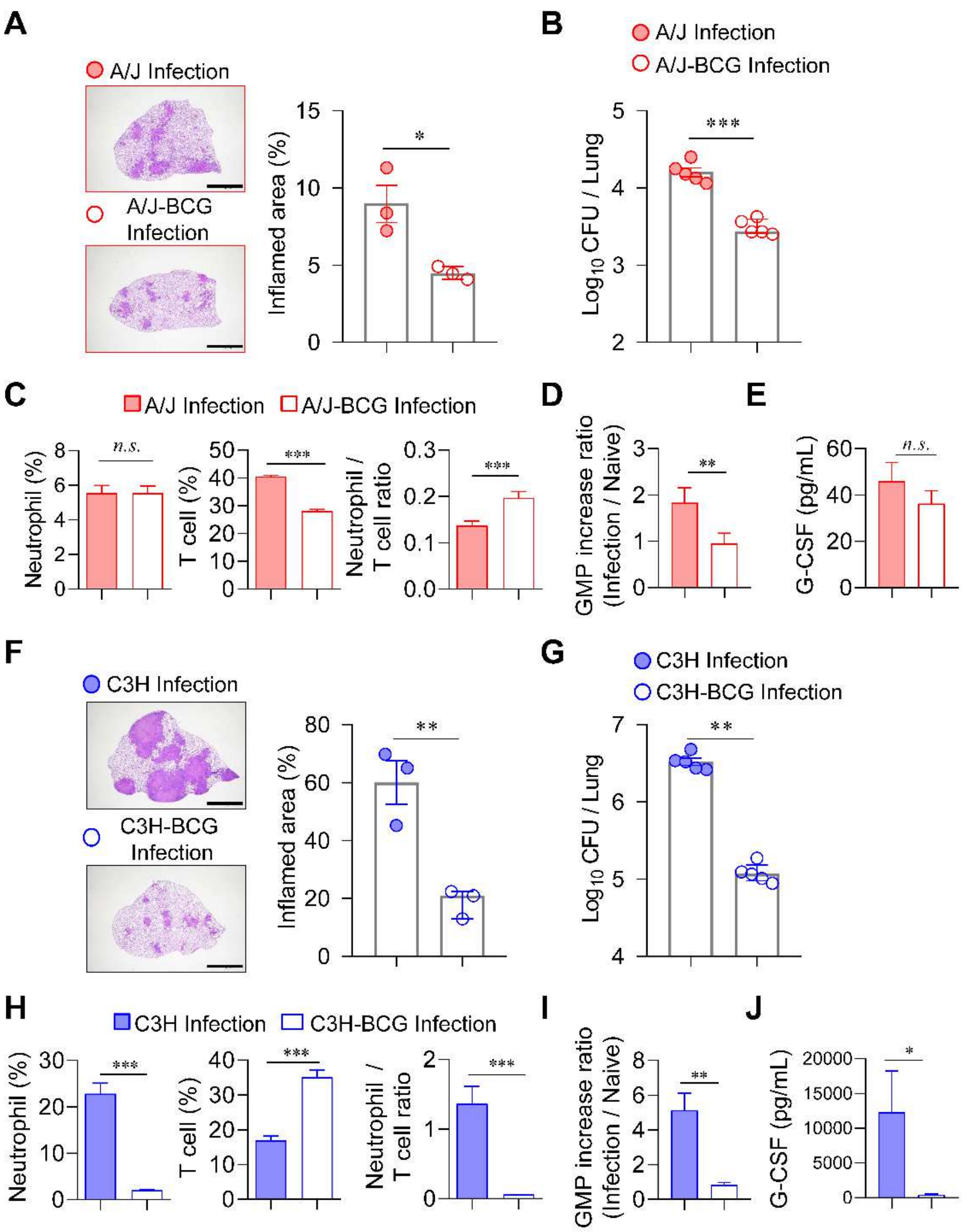

Figure S9. Association of normalized pulmonary neutrophil to T cell ratio by BCG vaccination with attenuated TB immunopathology in both TB-susceptible mouse strains. The A/J mice were vaccinated with BCG subcutaneously. After 10 weeks, the mice were challenged with Mtb K. After 4 weeks post-infection, (A) the lung histopathology was analyzed using H&E staining (scale bar = 2 mm), and (B) the lung bacterial burdens were assessed. (C) Neutrophil and T cell in the lungs were analyzed by flow cytometry. *n* = 4. (D) The GMP population of bone marrow cells were analyzed by flowcytometry. *n* = 3. (E) The G-CSF levels in serum were measured by ELISA. *n* = 3. (F) The C3H/HeJ mice were vaccinated with BCG subcutaneously. After 10 weeks, the mice were challenged with Mtb K. Lung histopathology was analyzed using H&E staining (scale bar = 2 mm), and (G) the lung bacterial burdens were

assessed. (H) Neutrophil and T cell in the lungs were analyzed by flow cytometry at 4 weeks post-infection. *n* = 4. (I) The GMP population of bone marrow cells were analyzed by flowcytometry. *n* = 3. (J) The G-CSF levels in serum were measured by ELISA. *n* = 3. Data are presented as mean ± SD. Statistical analyses were performed using one-way ANOVA with Tukey's multiple comparison test. *n.s.* = not significant. *$p < 0.05$, **$p < 0.01$, ***$p < 0.001$. GMP, granulocyte-monocyte progenitor.
